# Assessing the long-term effectiveness of Nature-Based Solutions under different climate change scenarios

**DOI:** 10.1016/j.scitotenv.2021.148515

**Published:** 2021-11-10

**Authors:** Eulalia Gómez Martín, María Máñez Costa, Sabine Egerer, Uwe A. Schneider

**Affiliations:** a Climate Service Center Germany (GERICS), Helmholtz Center, Chilehaus, Eingang B Fischertwiete 1, 20095 Hamburg, Germany; b Research Unit Sustainability and Global Change (FNU), University of Hamburg (UHH), Grindelberg 5, 20144 Hamburg, Germany

**Keywords:** Nature-Based Solutions, System dynamics, Participatory modelling, Droughts, Aquifer, Climate change

## Abstract

Nature-Based Solutions (NBS) have been gaining importance in many European cities to reduce floods' impacts. However, evidence of their effectiveness in reducing the impacts of droughts in rural areas are scarce. Besides, ignoring future climate conditions or the specific socio-economic context in which NBS is applied could decrease their long-term effectiveness. This study aims to stress the importance of developing scientifically-based and customised information on climate change impacts as a precondition for designing and implementing NBS. For that, a System Dynamic model was developed to analyse and understand the dynamic behaviour of NBS responding to different scenarios of climate change and socio-economic contexts. This article recognises the proactive involvement at all societal levels as an essential component to enhance and maintain ecosystem resilience and, therefore, NBS^1^effectiveness. Thus, participatory modelling activities were carried out to engage stakeholders in the model development process to obtain relevant bottom-up information and organise stakeholders' collective knowledge in a graphical structure that captures the system's main dynamics. The Medina del Campo Groundwater Body was used as a frame for the analysis. The study results highlight the need for developing scientifically-based and customised information on the impacts of climate change on NBS as an essential precondition to maintain their long-term effectiveness.

## Highlights

• Nature-Based Solutions are inherently multidimensional.
• Stakeholders knowledge should have real impact on the NBS assessment process.
• NBS may not be sufficient to adapt to an intense climate change scenario.
• Climate information is an essential precondition to design adaptable NBS.

## Graphical abstract

**Figure:**
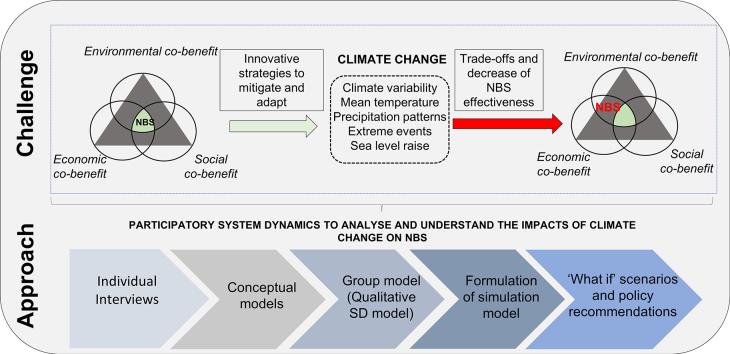

## 1 Introduction

The exponential population rise and the consequent growing demand for food in the next few decades are pressuring the agricultural sector into a more land-intense food production system (Panagopoulos and Dimitriou, 2020; Peter et al., 2017). Besides, the increasing transformation of natural landscapes into agricultural land is threatening further ecosystems degradation and destruction. Rural communities are burdened by multiple socio-economic stressors such as rapid shifts in agriculture markets, low accessibility to services and job opportunities, lower human development, and the overlooking of policymakers (G J S Sonneveld et al., 2018; Masson-Delmotte et al., 2018).

Additionally, climate change impacts on ecosystem processes are expected to compromise important regulatory ecosystem services such as waste-water treatment or erosion prevention. For instance, higher incidences of extreme precipitation events will increase soil erosion, affecting soil productivity and water retention capacity of soils threatening food security and agricultural incomes. (IPCC et al., 2018; Malhi et al., 2020). All these factors make rural communities particularly vulnerable. For this reason, migration patterns to cities are increasing, causing the abandonment of agricultural land and continuous loss of cultural landscapes (Pedroli et al., 2018).

Several governmental bodies have highlighted the need to shift to more sustainable food systems. New policy frameworks and climate adaptation actions are encouraged, which jointly address water and food security, preservation of agricultural and rural landscapes, mitigation of and adaptation to climate change, and conservation of ecosystems and biodiversity (UN Climate Action Summit, 2019; Global Commission on Adaptation, 2019; UN-REDD Programme, 2008, European Commission, 2015 or European Green Deal, 2019).

Among all the proposed strategies, the concept of Nature-Based Solutions (NBS) has occupied the EU political foreground in the past years (IUCN, 2016; Nesshöver et al., 2017; Somarakis et al., 2019). NBS include all actions and management strategies aimed at maintaining and enhancing ecosystem function while providing social and economic benefits. NBS highlights the role of biodiversity and sustainable use of natural resources to address food security, poverty, water scarcity, climate change, or other societal challenges. NBS are increasingly seen as strategic opportunities to simultaneously address climate and biodiversity crises. NBS have been applied worldwide to enhance the adaptive capacity of ecosystems and society to climate change while supporting the development of a more sustainable and resilient economy (Eggermont Hilde et al., 2015; Maes and Jacobs, 2017). The effectiveness of NBS in the future may be reduced by changes in species distribution, habitat fragmentation, biodiversity loss or increased climate variability (Seddon et al., 2020). Besides, the intensity of climate-related risks may increase in the next decade exceeding the capability of NBS to cope with these risks. Additionally, ignoring future climatic conditions or the specific socio-economic context in which NBS are applied could lead to ‘maladaptive’ practices (Seddon et al., 2019; Turner et al., 2020). For instance, afforestation projects that use fast-growing monocultures to increase carbon sequestration may be more vulnerable to climate change impacts, such as droughts, diseases, pests or fires. This could lead in turn to negative consequences, including stored carbon release into the atmosphere or water scarcity intensification in arid or semi-arid regions (Zhu et al., 2011).

Despite the growing evidence base for NBS to support climate change mitigation and adaptation, most studies investigating NBS effectiveness are limited to empirical studies, thus ignoring factors that cannot be studied empirically, i.e. long-term climate change projections. For this reason, scenario modelling approaches considering temporal projections of climate are crucial to understand the challenges and limitations of NBS in the context of environmental change.

Additionally, adaptation and mitigation strategies, including NBS, are often approached from an expert-driven, single-focus and top-down perspective. However, NBS affect a wide range of stakeholders including NGOs, national and local governments, scientists or civil society members.

Moreover, even though many biodiversity-based measures have been implemented in rural areas to support the development of sustainable agriculture, several authors have pointed out that the majority of NBS studies are specific from urban settings (Nesshöver et al., 2017; Solheim et al., 2021). For this reason, most of the indices and evidence developed to assess the long-term effectiveness of NBS are urban-specific. Thus, business models and financial instruments designed to encourage the mainstreaming of NBS into EU policies are also oriented for cities (Faivre et al., 2017).

In response to these gaps, we have adopted a system perspective and a multi-sectoral approach to examine the socio-economic dynamics associated with the implementation of NBS in the Medina del Campo Groundwater body (MGWB). MGWB is located in North-West central Spain, where the rural development and the regional economy depend strongly on agriculture. The uncontrolled overexploitation of the Medina del Campo aquifer has decreased groundwater levels and deteriorated the aquifer-dependent superficial ecosystems. Besides, climate change is likely to increase the frequency and intensity of droughts, threating further agricultural productivity, biodiversity and regional landscape. Therefore, the main aim of this study is to assess the potential of NBS to conserve and enhance water resources management without compromising agricultural productivity. For that, we developed a system dynamics model (SD) to analyse the long-term effectiveness of NBS strategies under different scenarios of climate change.

System dynamics (SD) is a mathematical modelling technique to mimic and understand the non-linear behaviour of complex systems (Voinov and Bousquet, 2010). SD has been used worldwide to understand a variety of socio-economic problems and to study the effect of various policy interventions (Balali and Viaggi, 2015; Santos et al., 2019). Although SD is not an optimal tool to exactly predict future system states, it is an effective method to indicate how different policy interventions may alter the tendency to move forward to a future state by simulating “what if” scenarios. Moreover, the graphical representation of SD facilitates the engagement with stakeholders in the model development process. We believe that NBS should integrate scientific and local knowledge. Accordingly, NBS should be understood as an inter-and transdisciplinary approach with multi-stakeholder engagement at the very heart of NBS implementation (Sonneveld et al., 2018). For this reason, a participatory modelling approach has been used to co-design the SD model.

This paper purposes an innovative method to analyse the long-term effectiveness of different NBS strategies by integrating regional downscaled climate ensembles for three RCPs scenarios into a quantitative system dynamics model. Participatory modelling techniques were used to obtain relevant bottom-up information and to facilitate the co-design process of the SD model, focusing on the stakeholder's perception on the main dynamics of the Medina del Campo System.

The co-development of a system dynamic model along with key stakeholders had four main objectives for this investigation. Firstly, to expose the causes of different system behaviours by analysing the complex network of interactions between physical, ecological and socio-economic factors affecting NBS effectiveness. Secondly, to observe the implementation of different NBS strategies under different input conditions (i.e. climate projections data). Thirdly, to anticipate possible rebound effects or policy resistance and identify suitable NBS strategies to be implemented. Finally, to engage with stakeholders at the beginning of NBS implementation to increase NBS effectiveness by promoting awareness and motivation of those taking part in the decision-making processes, thus providing a platform for the joint-ownership of results.

## 2 Methods and model development

### 2.1 Case study

**Fig. 1:**
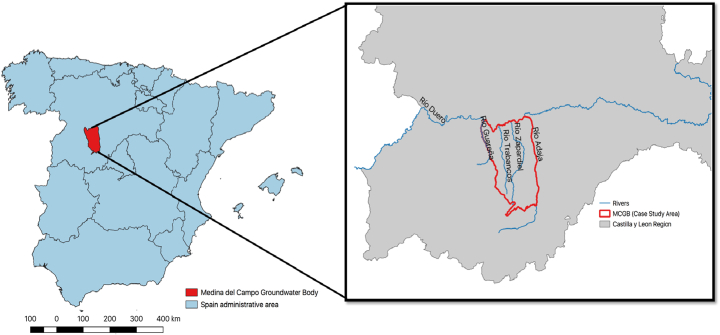
Groundwater body of Medina del Campo.

The Medina del Campo Groundwater body (MCGB), located in the Duero River Basin, north west-central Spain, forms the case-study for this region (Fig. 1). The MCGB covers an area of 3.700 km^2^ bordered by the Duero river to the east and the Sierra de Gredos and the Adaja and Trabancos rivers to the west. The rural development and the regional economy depend strongly on agriculture. Irrigated agriculture represents about 19% of the total agricultural land. Groundwater (GW) is the primary source of irrigation; 95% of total GW extractions are due to irrigation in agriculture. GW overexploitation has resulted in an uncontrolled decrease of the piezometric levels. Measurements show that GW levels have declined up to 20 m in some areas in the last 40 years. Simultaneously, the decrease of groundwater levels has increased their lithologic arsenic concentrations. Besides, excessive agricultural fertilization has spread nitrate pollution in the aquifer and superficial ecosystems.

These issues have caused a chain reaction resulting in severe deterioration of the main GW dependent ecosystems such as wetlands, rivers, riversides and streams. Many rivers and streams experience longer dry periods than before. More frequent and intense droughts have altered the region's landscape and biodiversity (Llorente et al., 2018).

Climate change impacts are likely to increase climate variability and extreme weather events such as heatwaves and heavy rainfalls (IPCC, 2014). The continuing environmental degradation and consequent loss of essential ecosystem services may reduce the resilience of the whole socio-ecological system. Additionally, environmental deterioration has increased the vulnerability towards climate-related risks such as drought, floods or heat waves and has impacted livelihoods, human security and well-being.

### 2.2 System dynamics modelling approach

The System Dynamic approach was described for the first time in 1950 by Forrester and Cole (J.W. Forrester, 1958). Since then, it has been applied in several disciplines such as economics, environmental studies or social sciences. Several studies have used System Dynamics (SD) to address complex problems such as water scarcity (Stave, 2003; Sušnik et al., 2012), flood risk reduction (Pagano et al., 2019) or health care system management (Homer and Hirsch, 2006). The theoretical concepts of system dynamics are grounded in systems theory, information science, organisational theory, control theory, tactical decision-making, cybernetics and military games. SD is a mathematical method that facilitates the description of systems behaviour by analysing the feedback structure of complex systems. The central aspect of this approach is the observation of the relationships between the different elements of the system (Sterman, 2000). Understanding the structure of these relationships can help to prevent undesirable consequences arising from various policies and actions. An essential premise in SD is that dynamic behaviour is a consequence of the system structure. Several interactions linked by feedback loops compose systems. The combination of feedback loops with delays and non-linear relationships produce a wide variety of behavioural characteristics.

Mathematically, the SD approach is based on linked first-order differential (or integral) equations that are represented in the simulation model in the form of stocks and flows. Stocks are accumulations within the system (i.e. amount of groundwater in the aquifer) and are represented by rectangles in the model. They represent the (observed) state of the system that can be changed by flows. Flows may be inflow to a stock or an outflow from stock. They are represented by pipes with valves controlling the rate of flow. In this study, the development of a simulation system dynamic model has provided several advantages. Firstly, it has allowed the integration of quantitative and qualitative data from different sources and spatial-temporal scales. Secondly, although SD is not an optimal tool to exactly predict future system states, it is an effective method to indicate how different policy interventions may alter the tendency to move towards any future state by simulating “what-if” scenarios (Simonovic, 2002). It was possible to observe how the implementation of different NBS strategies under different input conditions (i.e. climate change scenario) affected the behaviour of the system as a whole. Finally, the conceptual representation of the model in a graphical structure facilitated the engagement of stakeholders in the co-design of the SD model allowing the integration of knowledge of end-users and stakeholders within the model.

**Fig. 2:**
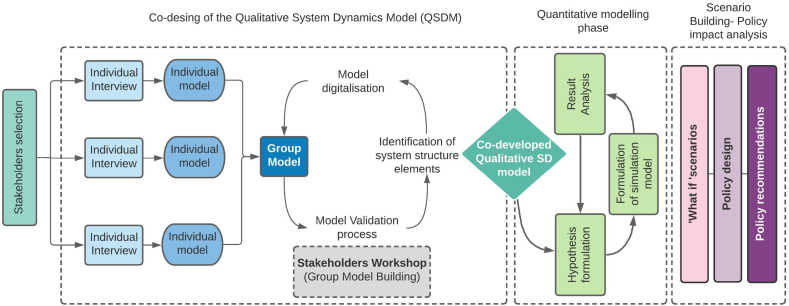
Overview of the modelling framework carried out in Medina del Campo Groundwater Body.

In this study, the development of the computer-simulated model started from a participatory modelling phase. Stakeholders input was used to develop a Qualitative System Dynamics Model (QSDM) representing key variables and relationships within the system. The qualitative model developed with stakeholders was used to develop the dynamic hypotheses before the quantitative stock-flow model was developed. The methodological process adopted is schematized in the following Fig. 2.

#### 2.2.1 Participatory Modelling approach

The implementation of participatory modelling techniques allows for the integration of end-users and stakeholder's knowledge with scientific analysis, e.g. with development and application of scientific models. It has proven to be a suitable methodology to engage non-scientists with scientists as it introduces a new perspective for both the modeller and the stakeholder (Reed et al., 2009). Stakeholder's involvement from an early stage of the process increases their sense of ownership in the model. Furthermore, it enables participants to have a better understanding of the model, increasing their confidence in the outputs provided (van den Belt, 2009). Additionally, participatory modelling techniques promote information exchange and the collective and individual learning of those taking part (Vennix, 1999). Participatory modelling allowed us to obtain data coming from formal and non-formal sources as well as the analysis of multiple decision drivers and their interactions. Moreover, we were able to identify critical feedbacks and leverage points in order to support the process of NBS implementation (Voinov and Bousquet, 2010).

During the exercise, stakeholders identified essential factors and issues that were relevant for modelling the system. Additionally, modelling components needed to assess the long-term effectiveness of NBS were identified. The identification was carried out after analysing the causal connections and influences involving the multiple dimensions of NBS, ranging from economic, social and environmental factors.

The participatory modelling exercise was divided into two phases (see Fig. 2). Firstly, individual semi-structured interviews were carried out along with conceptual mapping techniques. In each interview, individual conceptual models were created in a co-design process. The interviews used a semi-structured format designed to avoid straightforward questions and answers, maintaining an engaging debate among the stakeholder and the interviewer. A syntactic rule was used to differentiate the social, economic and environmental elements in order to facilitate the development of the conceptual model. The objective of the activity was to conceptualise and understand the socio-environmental system in which the NBS will be applied; more specifically, stakeholders identified key factors and issues relevant to the modelling of NBS. For example, governance and socio-economic structure, weather-related risks or potential barriers and issues associated with NBS. Secondly, the information collected in all individual interviews was combined to develop a group model (GM). The GM was built by combining the information gathered in all individual models. The GM contained the variables and relationships that commonly appeared in the individual models. Before starting the aggregation process, a language homogenisation phase was needed since some variables were described by stakeholders using different terminologies. To avoid overcomplexity, the variables and relationships that were not repeated in most individual models were eliminated (Ryan et al., 2021; Williams et al., 2019). This GM was validated in a group model exercise (GMB) which was carried out in a two-hour long workshop. The GMB exercise was used to increase the communication and to facilitate the consensus agreement process and the shared vision among stakeholders. During the validation process, the conceptual model was presented and discussed with stakeholders in a one-day workshop. In contrast to the individual interview, the system was analysed from a collective perspective.

The participatory modelling process resulted in the development of a Qualitative System Dynamics Model (QSDM) (see Appendix A of the Supplementary material). The QSDM was used as a graphical tool to represent the feedback structure responsible for the cause of dynamics of the Medina del Campo system. The QSDM set the basis for the quantitative system dynamics model used to analyse the added value of Nature-Based Solutions as well as their long-term effectiveness. It was used to define the model boundary as well as the elements that should be included in the model (elements that influence the system's behaviour).

For a more detailed description of the participatory modelling process carried out in Medina del Campo as well as for the qualitative analysis of the QSDM see Gomez et al. (2019).

#### 2.2.2 Quantitative modelling phase

QSDMs do not distinguish between stocks and flows, meaning that the accumulation of resources in a system, as well as the rates of change that alter those resources, are not visible. A stock is a measurable accumulation of physical or non-physical resources (e.g. amount of water in aquifer or level of information/citizens awareness). It characterises the state of relevant variables in the system, storing the memory of their state at previous time steps, thus enabling description of their evolution. Flows affect the state of stocks via inflows and outflows, thus supporting interconnections among the variables within a system. For example, the change of the state of aquifer level depends on rain (inflow) or water extractions (outflow). Mathematically, stocks integrate their net flows; the net flows are the derivative of stocks. Identifying and modelling the behaviour of stocks is crucial to analyse the dynamic evolution of the essential variables and potential mutual influences (Sterman, 2000).

Given the interdisciplinary and complexity of NBS, a modular structure was adopted to quantify the SD model. Different modules coming from different disciplines were linked together by mutual feedbacks.

The validation of the SD model was done in two steps. Firstly, the qualitative SD model was validated in a stakeholder's workshop. The essential premise in SD is that the structure of the system determines the behaviour of the different elements. Therefore, validating the model structure with key stakeholders increases confidence.

Secondly, the quantitative model was simulated with historical data of precipitation and evapotranspiration (time-period 1979–2012). The simulation results show a decreasing trend of the Medina del Campo aquifer levels, going in concordance with the results of other studies (Borowiecka et al., 2019; Faneca et al., 2019; Lopez-Gunn et al., 2021).

### 2.3 Description of the model

**Fig. 3:**
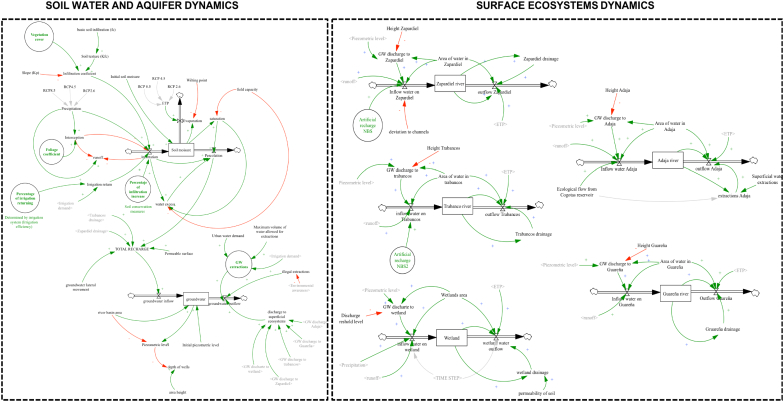
Environmental sub-model describing soil water and aquifer dynamics as well as surface ecosystems dynamics. Arrows in green representing positive polarity; red arrows representing negative polarity. Stocks are indicated with rectangular boxes. Green circles represent potential areas of intervention (NBS).

**Fig. 4:**
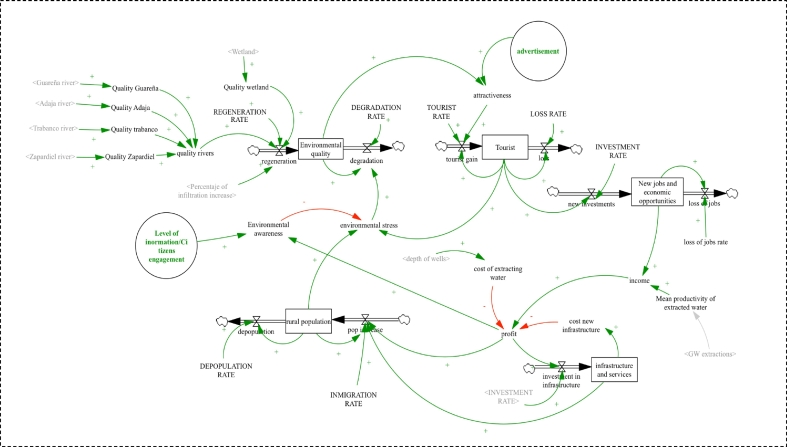
Socio-economic sub-model describing soil water and aquifer dynamics as well as surface ecosystems dynamics. Arrows in green representing positive polarity; red arrows representing negative polarity. Stocks are indicated with rectangular boxes. Green circles are potential areas of intervention.

The system dynamics model represented in Fig. 3, Fig. 4 was developed using VENSIM [30] software. The Qualitative System Dynamics Model co-developed with stakeholders was used to define the overall structure of the model (see Appendix A). The grounds behind the individual sub-models and their fundamental dynamics are addressed in the manuscript. At the same time, the full list of equations and parameter values are included in Appendix B of the Supplementary material.

The grey variables in the model describe the principal connections between different sub-models. These variables help to identify relationships and influences that are often difficult to recognize. The polarity of the relationship was represented by a green arrow (positive polarity) or red arrow (negative polarity). The polarity is used to indicate how the dependent variable changes when the independent variable changes.

The model is run for 100 years allowing the long-term assessment of NBS effectiveness under different projected climate change scenarios. The time step of the simulation is one month. To facilitate the simulation of groundwater control measures, the model assumes that water extractions are constant over the year. The main dynamic assumptions of each sub-model are summarised in the following:

#### 2.3.1 Environmental sub-model

Based on the knowledge obtained from the individual interviews, the environmental sub-model (Fig. 3) assumes that the water is stored in three different storage levels, soil water, groundwater and superficial ecosystems (Trabancos river, Guareña river, Adaja river, Zapardiel river and wetlands). The water entering into the soil moisture layer depends on the precipitation fraction that infiltrates into the soil (infiltration coefficient), the monthly retention of rainfall in the foliage (interception), and the fraction of irrigation water that returns into the system (irrigation return) (Schosinsky, 2006).

The infiltration coefficient is dependent on the slope, the vegetation cover and the basic soil infiltration (fc). The fc is a characteristic of the soil that corresponds to the permeability of the soil when it is saturated. The model assumes that the lower the slope of the land and the more prominent the vegetation cover, the higher the percentage of rainwater which infiltrates (IGME, 2008; Schosinsky, 2006). Soil conservation (SC) measures such as mulching, crop cover or no-till practices have proven to increase water infiltration up to 40%. The model assumes that increasing infiltration by a certain percentage (depending on the conservation measure), allows the estimation of the impact of SC in the system.

The vegetation leaves retain a portion of the rain, preventing water from reaching the soil. The interception is a function of the foliage coefficient which differs depending on the type of vegetation cover. For example, a forest may intercept 40% of rain while crops such as alfalfa can intercept between a range of 10 and 35% of the total rainfall.

The irrigation return is defined by the irrigation efficiency (percentage of irrigation returning) determined by the type of irrigation system used in agriculture—highly efficient irrigation methods such as drip irrigation use virtually 90% of the irrigated water (Sithole et al., 2019; Thierfelder and Wall, 2010). The model assumes that 10% of the remaining water constitutes the irrigation return into the system. This means that the higher the irrigation efficiency, the lower the irrigation return.

The monthly rain that is not retained by the vegetation foliage or infiltrated into the soil flows away in the form of surface run-off to the superficial ecosystems (in the model, wetlands and rivers).

The amount of water stored in the soil level (stock) depends on the amount of water that infiltrates (explained above) and the amount of water that evaporates and percolates into the aquifer. Both processes depend on the field capacity (maximum accumulation of water that an unsaturated soil can have) and the wilting point (minimum humidity to maintain vegetation). The model assumes that when the water stored in the soil equals the wilting point, the evapotranspiration stops, as it assumes that the plants close their stomata (Schosinsky, 2006).

The percolation is described in the model as a function of saturation. The maximum percolation capacity occurs when the soil reaches its field capacity (100% saturated).

The total recharge (groundwater inflow) is determined by the amount of water that percolates, the underground run-off coming from other groundwater bodies (groundwater lateral movement in the model) and water drainage from superficial ecosystems (Trabanco and Zapardiel river). The model assumes that the groundwater lateral movement is constant over the year. It also implies that rivers drainage into the aquifer is a constant percentage of the monthly river flow.

Based on the individual interviews and the discursion generated during the group model building activities, the groundwater extractions and the discharge to superficial ecosystems constitute the outflow of the groundwater level. GW extractions include legal and illegal extractions. Legal extractions are regulated by the irrigation demand and the maximum volume of water allowed for extractions. Illegal extractions are a function of environmental awareness (defined in the socio-economic sub-model). An increase of environmental awareness results in a reduction of the illegal extractions.

The decrease of the piezometric level (level to which water is confined in an aquifer) occurs when the total recharge is lower than the GW extractions. If the piezometric level is less than a certain level, the surface ecosystems disconnect from the aquifer. On the contrary, if the piezometric level reaches a certain threshold, water flows to the surface ecosystems.

Following stakeholder's perception on the Medina del Campo system, the superficial ecosystems are represented in the model with five stocks, the four most important rivers in the region (Adaja, Trabancos, Guareña and Zapardiel) and one stock representing the currently-dried wetlands.

The model assumes that the only waters that flow into these levels are the surface run-off and the groundwater discharge into superficial ecosystems. GW discharge depends on the piezometric level and the height at which the ecosystem is in the region. The total run-off entering into each of the ecosystems depends on the river/wetland catchment area. The model includes an additional inflow variable for Trabancos and Zapardiel river. This variable can be activated to simulate an artificial recharge of the aquifer through surface techniques. The model assumes that when the artificial recharge variable is activated an additional 0.77Hm3 of water will flow into the Zapardiel river and 0.6 Hm3 will flow into Trabancos.

Evapotranspiration and drainage water into the aquifer are the only variables considered to calculate the outflow of water.

##### 2.3.1.1 Climate data integrated in the environmental sub-model

The climate component has been highly relevant for this study as the intention is to analyse the performance of NBS strategies under different climate change scenarios. Therefore, climate projection information for three Representative Concentration Pathways scenarios (RCP 2.6, RCP 4.5 and RCP 8.5) has been integrated into the SD model. Specifically, monthly precipitation and monthly mean temperature were obtained from the EURO-CORDEX ensemble (Jacob et al., 2020). RCPs describe future climate scenarios based on possible volumes of greenhouse gases emitted in the next century. According to the IPCC, RCP 2.6 requires that carbon dioxide (CO2) emissions decline from 2020 to zero at the end of 2100; RCP 4.5 is described as an intermediated pathway in which CO2 emissions peak at 2040 before declining; RCP 8.5 describes a future scenario in which emissions continue to rise through the next century (van Vuuren et al., 2011).

Climate simulations for past-time periods (hindcasts) from different models were compared with observational data. The comparison was made plotting both time series (for precipitation and temperature) and comparing the mean and the standard deviation. The objective was to select the model that most accurately represented the real-world climate system of MCGWB. Finally, the climate projections data used was generated by the driving model MOHC-HadGEM2-ES and downscaled with the Regional climate model (RCM), KNMI-RACMO22E. See Appendix C in the Supplementary material for more detailed information about the climate models that were compared.

After the appropriate model was selected, time series of precipitation and evapotranspiration (time-period 2019–2100) were integrated into the SD model.

Potential Evapotranspiration was calculated following the equation developed by Blaney & Criddle (ONU, 1972) and expressed in Eq. (1).

(1) $$\mathrm{ETP}=\left(8.10+0.46T\right)\times \mathrm{Ps}.$$

Where ETP is potential evapotranspiration in units mm/month; T is mean monthly temperature (^0^C/month), and Ps corresponds to the percentage of hours of monthly sunlight compared with the annual mean of sunlight.

Certain parameters of climate models carry considerable uncertainty depending on the climate model used. Thus, it is difficult to determine the actual effect that they have on the final results. It is necessary to use multi-model ensemble simulations to show the uncertainty range produced by climate models.

The main aim of the SD model presented in this article is to provide a valid description of the essential system structure and the resulting system dynamics; it does not intend to provide detailed quantitative results. For this reason, multi-model ensemble simulations were not carried out in all simulation scenarios.

However, a climate model-ensemble was used for the two climate variables (precipitation and evapotranspiration) in order to show the uncertainty range produced by climate models. This approach was also used for the variable “piezometric level” in the BAU scenario. Piezometric level was considered as the main indicator to describe the state of the aquifer. For this reason, a multi-model ensemble was used in this variable to highlight the need to consider climate uncertainty ranges in NBS decision-making.

#### 2.3.2 Socio-economic sub-model

Based on Bossel's Systems Zoo (Bossel, 2007), the socio-economic sub-model contains five state variables, environmental quality, tourists, new jobs and economic opportunities, infrastructure and services and rural population (Fig. 4). These represent qualitative variables which are relative and dimensionless (ranging from 0 to 3) in order to understand the qualitative dynamics of this sub-system. The quality of the environment changes by natural regeneration and degradation of the environment. Natural regeneration increases when the quality of superficial ecosystems increases. The model assumes that the amount of water determines the level of ecosystems quality. However, it has been considered that the environment needs time to regenerate; for this reason, the model includes a delay in the regeneration rate. Environmental degradation occurs due to the environmental stress caused mainly by the increase of tourism in the area and by the rural population. The number of tourists decreases by tourist loss and increases by tourist gain. Tourist gain depends on the attractiveness of the area. Attractiveness is a function of environmental quality and advertisement (level of promotion to attract new tourists).

The model assumes that the higher the environmental quality and advertisement, the higher the gain in tourists. A delay function was included in the tourist gain rate to indicate a postponed effect from the increase in environmental quality to the increase in tourists.

The increase in tourists has a positive effect on the creation of new jobs and economic opportunities which, simultaneously, has a positive effect on income (money received by locals). The income is also produced with the use of the water extracted from the aquifer. Therefore, the more water is withdrawn from the aquifer, the higher the income. Income has a positive effect on profit which is a function of income, cost of investing in new infrastructures and cost of groundwater extractions—the cost of extractions increases as the depth of the wells increases (when the piezometric level declines).

It is considered that the economy of the area is a critical aspect to maintain the rural population. For this reason, the model assumes that an increase in profit leads to an increase in the rural population. The increase in services and infrastructure also positively affects the increase in the rural population. Environmental awareness can regulate the environmental stress caused by tourists and the local population. However, it is assumed that this regulation requires a specific time to produce effects on environmental stress. The model simulates this phenomenon using a delay function. The level of environmental awareness is defined by the level of information and citizens engagement as well as by the level of the economic growth.

### 2.4 Scenario building - dynamic hypothesis

Five scenarios have been simulated to ‘test’ the impact of soil conservation practices (NBS) and groundwater extractions control measures on aquifer levels (summarised in Table 1). Practices such as crop rotation, tillage reduction, mulching or cover cropping have proven to increase soil infiltration up to 30% by preventing soil degradation and building organic matter (Sithole et al., 2019; Thierfelder and Wall, 2010). From the modelling point of view, NBS implementation is represented by an increase of 20% of the total infiltration rate (see Fig. 3). Additionally, it is expected that the implementation of NBS delivers other benefits such as biodiversity support or improvement in water quality. The model describes this improvement by increasing 20% environmental quality (Fig. 4).

**Table 1:**
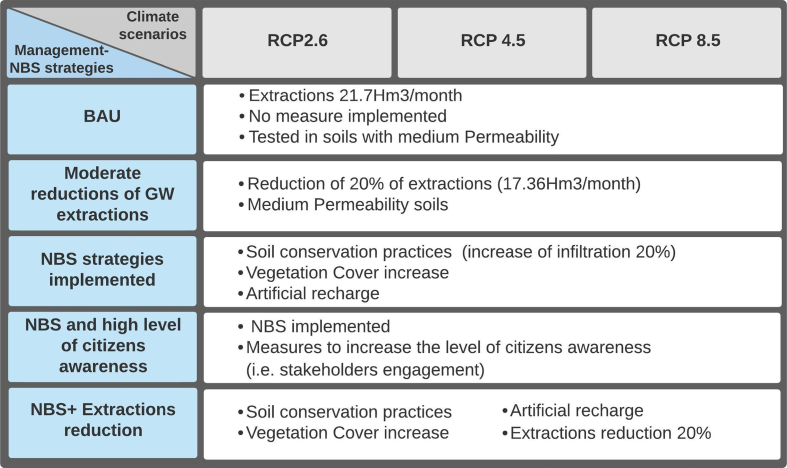
Scenarios simulated under different management/NBS strategies and RCP scenarios.

The implementation of NBS focused on conserving and restoring grasslands have been represented in the model by increasing vegetation fraction (Fig. 4).

Measures to control groundwater extractions have been represented in the model by reducing the “maximum water volume allowed for the extractions” variable (Fig. 3).

Each strategy (NBS and groundwater extractions control) has been simulated for three RCPs (RCP2.6, RCP4.5 and RCP 8.5) to assess their long-term performance. The initial dynamic hypothesis is that implementing NBS in the MCGW may benefit both levels, environmental and socio-economic. Climate change may impact ecosystems function; thus, compromising NBS capability to produce benefits (co-benefits). Therefore, NBS multifunctionality may be lower in scenarios where climate change is more intense (i.e. RCP4.5 and RCP8.5). Consequently, the long-term effectiveness of NBS may be compromised in a climate change context.

## 3 Results

The following section presents the main results and research findings. The analysis of the dynamic behaviour of four state variables (piezometric level, environmental quality, new jobs and green opportunities and rural population) has been used to analyse the long-term performance of NBS in the economic, social and environmental sustainability dimensions.

**Fig. 5:**
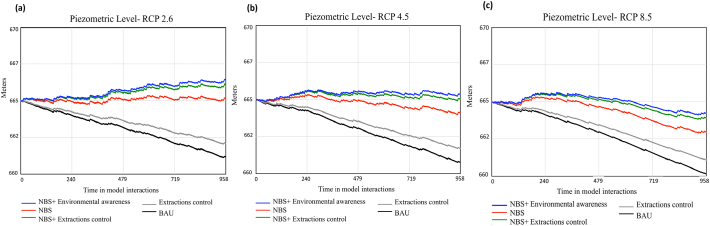
Dynamic behaviour of Piezometric level state variable under five different management scenarios and RCPs.

Fig. 5a, b and c show the dynamic behaviour of the piezometric level under five different management and climate change scenarios. The results show a continuous decrease in the aquifer levels for the BAU scenario (no-measures). The decrease shown in RCP 8.5 (Fig. 5c) is more pronounced than scenario RCP2.6 (Fig. 5a). Although reducing GW withdrawals by 20% diminishes the tendency of aquifer levels to decrease, it is not enough to change the trend, and the levels continue to decrease for the three RCPs. The results show an improvement in the levels of the aquifer for RCP2.6 when NBS are applied. Contrarily, this improvement cannot be observed for the scenarios of RCP 4.5 and 8.5, where the levels continue to decrease. This decrease is slightly less significant than in scenarios where no NBS are applied.

The most notable increase of the aquifer level is shown when applying NBS and promoting environmental awareness. The improvement of the aquifer levels is produced due to the reduction of illegal extractions. An improvement of the piezometric level is also observed when NBS are applied with GW extractions control. On the one hand, NBS measures focused on improving soils quality, increase the total recharge of the aquifer. On the other hand, measures such as limiting the maximum volume of GW extractions or changing the crop-type to other less-demanding crops, reduce total GW extractions. Although an increase of the piezometric level is observed for RCP2.6, the increase is not enough to reach the basin of the superficial ecosystems preventing the aquifer-superficial ecosystems reconnection.

This aquifer level improvement is maintained for the RCP4.5 scenario. However, the results show that in the long-term, these measures would not be sufficient in RCP 8.5, where more investments in adaptation measures will be required.

**Fig. 6:**
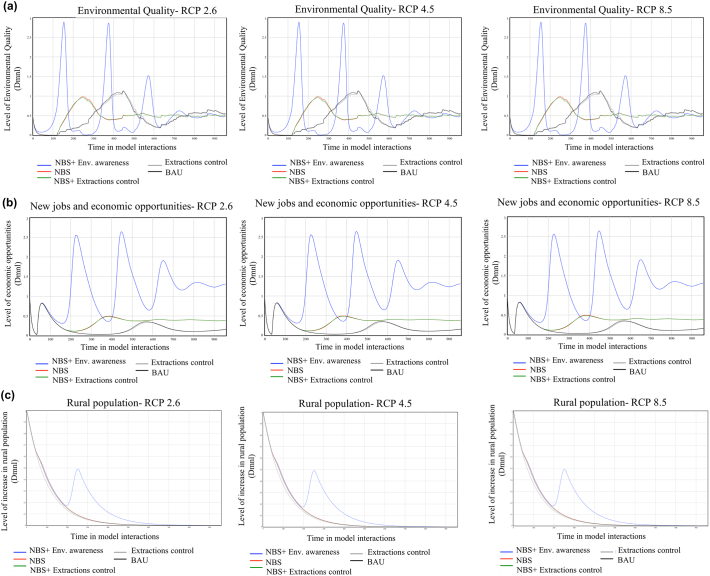
Environmental quality (A), new jobs and economic opportunities (B) and rural population (C) under different management and RCP scenarios. The results are dimensionless and relative in a scale ranged from 0 to 3. Being 3 the higher value.

Fig. 6A shows the dynamic behaviour or environmental quality (EQ) state variable under different management scenarios and three RCPs. The results show no significant difference between the different RCP scenarios. The lack of variation between the three RCPs is explained because environmental quality strongly depends on the superficial ecosystems state. The simulation results show that reconnection of aquifer-superficial ecosystems is not achieved in any of the RCPs scenarios. The influence of several balancing loops with delays present in the socio-economic sub-model produces an oscillating behaviour of the environmental quality variable for all management scenarios. This oscillation is especially noticeable when NBS is combined with measures to increase environmental awareness. Even though the initial condition describes a strong citizen engagement level, environmental quality reduces drastically due to the pressure suffered by the increase in tourists and rural population. As the environmental quality diminishes, so does the environmental pressure produced by tourists since the area is less attractive; therefore, environmental quality increases again. This behaviour occurs until equilibrium is reached.

Combining NBS with extractions reduction produces an increase in environmental quality. However, this increase is less pronounced than in the scenario mentioned above (NBS and citizens awareness). Additionally, the positive effect of NBS in EQ requires more time to become visible. The initial increase in EQ produced by these measures (NBS + GW reduction) follows an EQ decrease. Reducing GW extractions may negatively affect the area's economic profit as it is highly dependent on irrigated agriculture. A decrease in profit leads to a decrease in environmental awareness and, thus, on EQ.

The same pattern of behaviour is followed by BAU and NBS-NCA (NBS without citizens awareness) scenarios.

Fig. 6B shows the dynamic behaviour of new jobs and economic opportunities state variable (NJEO). The model results do not provide evidence showing a discernible influence of climate change on creating new NJEO. Following the reasoning described above, there are no variations in behaviour among the three RCPs (Fig. 6b). However, different patterns can be observed within the management scenarios. The implementation of NBS and measures focused on improving citizen awareness (i.e. stakeholder's engagement, increasing level of information) manifests to be the most effective strategy to achieve higher levels of NJEO. Although implementing NBS and extraction control measures produces an initial increase of this state variable, a rapid decrease is produced in the following years. Therefore, it indicates a positive causal connection between environmental quality and new jobs and economic opportunities. The same pattern of behaviour is observed in the scenario where NBS are applied without any additional measures; thus, indicating a general positive effect of NBS on NJEO.

BAU and GW extractions reductions scenarios do not show any significant improvement in NJEO levels.

The model simulation results show a decrease of rural population on all management and climate change scenarios (Fig. 6C). Scenarios where NBS are implemented with measures to increase citizens awareness show one peak of increase followed by a rapid decline of rural population levels. The influence of NBS on environmental quality and the economy of the area (NJEO) is not sufficient to maintain the rural population.

The general results show that, although the implementation of NBS improves the state of the aquifer, it is not enough to achieve a full recovery. Likely, NBS implementation is not enough to address the main environmental, economic and social challenges.

**Fig. 7:**
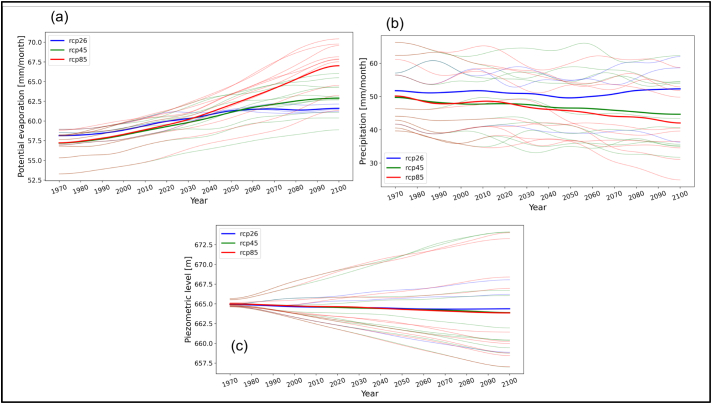
Multi-model simulation with two climate components of the system dynamic model (Potential Evapotranspiration and precipitation) and one auxiliary variable (piezometric level). The curves where smoothened using a Gaussian filter. Thin lines indicate the specific climate models, whereas thick lines show the model ensemble mean.

Fig. 7a and b show the multi-model simulation results for the two climate components that were included in the SD model, precipitation and potential evapotranspiration. Fig. 7c shows the multi-model simulation results for the piezometric level state variable (in the BAU scenario). The results show higher evapotranspiration in RCP 8.5 compared with RCP 4.5 and RCP2.6. A reduction of precipitation is also observed in RCP 8.5 and RCP 4.5. Although lower levels of piezometric levels are shown for RCP 8.5, the difference between the different RCPs is not significant. However, the results reveal a high uncertainty among the different climate models.

## 4 Discussion

Adaptation strategies, such as Nature-Based Solutions, are inherently multidimensional. However, NBS complexity is not often captured in research studies and frameworks. NBS are frequently framed in a reductionist way, focusing only on a subset of impacts and ignoring potential trade-offs among NBS and other system elements. For example, climate change impacts are frequently overlooked in NBS assessments. However, it is known that changes in mean temperature, species distribution or precipitation patterns are highly likely to alter ecosystem functions and, thus, NBS functionality. Besides, the long-term capability of NBS to deal with extreme weather events such as droughts may not be sufficient in a climate change context.

Additionally, a significant part of the NBS research studies has been contextualised in cities. Usually, the urban-centred evidence on NBS effectiveness cannot be transferred to rural contexts. Rural areas are affected by different pressures and socio-economic circumstances. In this research, we seek to assess the socio-economic dynamics associated with the implementation of NBS in rural areas by analysing the Medina del Campo Ground Water Body system. Special attention has been given to the long-term effects of these measures under different climate change scenarios.

We developed a system dynamics model (SD), starting from a participatory modelling phase to analyse the long-term effectiveness of NBS strategies under different scenarios of climate change. The intention was to a) analyse the main dynamics of the system; b) assess the effect of different climate change scenarios; c) see the pathways of development of the system depending on different policy measures to analyse NBS suitability; and d) engage stakeholders in the model development process by contributing to model assumptions and parameters.

We believe that stakeholder knowledge and priorities should have a real (not just superficial) impact on the model. Engaging stakeholders in the co-design of the SDM has provided multiple advantages. Firstly, it has supported constructive and targeted discussions relevant to identifying barriers and limitations of NBS implementation. Secondly, it has allowed for the integration of local knowledge in a graphical structure used to set the basis of the quantitative SD model supporting the integration of qualitative and quantitative data. Thirdly, complex interconnections among system elements were revealed, helping to anticipate possible policy resistances or rebound effects and suitable NBS to act on the system. Finally, the process itself has promoted awareness and collective learning of those taking part in the participatory modelling process.

The main advantage of SD over other approaches such as statistical modelling is gaining insights and understanding from data-poor problems. The basis of the SD approach is the recognition that the relationships among different system elements are often more important in determining systems behaviour as the individual components themselves. Therefore, the results are highly sensitive to assumptions, increasing uncertainty in results. The simulation of the quantitative SD model does not intend to provide detailed predictions on water level (piezometric level) changes due to NBS implementation. The model was used to study the different patterns of behaviour produced when implementing NBS or other management strategies (i.e. measures to increase environmental awareness, groundwater extractions control). It was possible to observe the potential of NBS to improve aquifer levels while delivering additional benefits (i.e. creating new job opportunities, improving environmental quality or reducing depopulation) in the Medina del Campo system.

The results show that the long-term effectiveness of NBS is highly dependent on the socio-ecological context in which NBS are implemented. For example, scenarios with a low level of environmental awareness limit the potential of NBS to deliver certain co-benefits (i.e. creation of new jobs and economic opportunities). Besides, this study also demonstrates that the implementation of NBS may not be sufficient to adapt to a worst-case climate change scenario (RCP 8.5). Therefore, additional measures such as controlling groundwater extractions or increasing environmental awareness by engaging stakeholders before implementing NBS are essential to maintain the delivery of NBS co-benefits. Additionally, we stress the importance of accounting for climate model uncertainty using multi-model ensemble simulations in NBS assessments. Ignoring uncertainty ranges, for example, by only using one climate model, does not allow informed decision-making. Although regional downscaled climate projections were used to analyse the long-term effectiveness of different NBS strategies, evaporation and changes in rain patterns have been the only climate change impacts considered. Soil degradation, plant pathogens, pests, or increase of ground-level ozone are just a few of many climate change impacts that could ultimately compromise the effectiveness of NBS in rural areas. Therefore, new methods and tools supporting the integration of climate change data with other socio-economic elements may contribute to the decision-making process of NBS design and implementation. The uncertainty associated with climate projections has several implications on NBS assessments that, if not correctly understood, may complicate and jeopardise the correct assessment of NBS performance. Climate uncertainty is difficult to quantify and calculate. However, the proper communication and management of this uncertainty represent an opportunity for NBS decision-making. Hence, new research strategies focusing on transforming climate data (and its associated uncertainty) into customised and tailored information to be used in NBS assessments and evaluations are needed.

## 5 Conclusion

Developing scientifically based and customised information on the impacts of climate change by assessing uncertainty ranges is an essential precondition to design and implement long-term effective and flexible NBS.

The results of this study show that the socio-economic context in which NBS are applied significantly influence the performance of NBS. The benefits delivered by NBS may be compromised if a balance between nature, economic growth and society is not found. NBS should not be considered as a single action to protect or restore nature but as a process that engages with society to merge natural and human systems into a wholly unique system.

The following are the supplementary data related to this article.

Appendix A Fig. A1 Qualitative System Dynamics Model co-developed with stakeholders. Highlighted in green the environmental variables and relationships, in blue the socio-economic elements are represented and in red the risks. The polarity of the relationship is represented by a positive (+) or negative (-) symbol. The processes or decisions that require some time to occur are indicated with a delay mark (//). Relevant feedback loops are numbered and their polarity and direction have been indicated: Reinforcing or positive loop (R), Balancing or negative loop (B). Appendix A

Appendix B Table B.1 List of equations and parameter values. Appendix B

Appendix C Appendix C. Table 1C. List of climate model used in the evaluation and multi-model simulations (Jacob et al., 2020). Appendix C

## CRediT authorship contribution statement

**Eulalia Gómez Martín:** Conceptualization, Methodology, Writing – original draft. **María Máñez Costa:** Conceptualization, Writing – review & editing. **Sabine Egerer:** Methodology, Writing – review & editing. **Uwe Schneider:** Writing – review & editing.

## Declaration of competing interest

The authors declare that they have no known competing financial interests or personal relationships that could have appeared to influence the work reported in this paper.
