# Supplementary material for: Assessing the long-term effectiveness of Nature-Based Solutions under different climate change scenarios
Source: Sci Total Environ. 2021 Nov 10;794:148515. doi: 10.1016/j.scitotenv.2021.148515 (PMC8434435; doi:10.1016/j.scitotenv.2021.148515)
Supplement: Appendix A — Fig. A1 Qualitative System Dynamics Model co-developed with stakeholders. Highlighted in green the environmental variables and relationships, in blue the socio-economic elements are represented and in red the risks. The polarity of the relationship is represented by a positive (+) or negative (-) symbol. The processes or decisions that require some time to occur are indicated with a delay mark (//). Relevant feedback loops are numbered and their polarity and direction have been indicated: Reinforcing or positive loop (R), Balancing or negative loop (B). [file mmc1.docx]

**Appendix A**


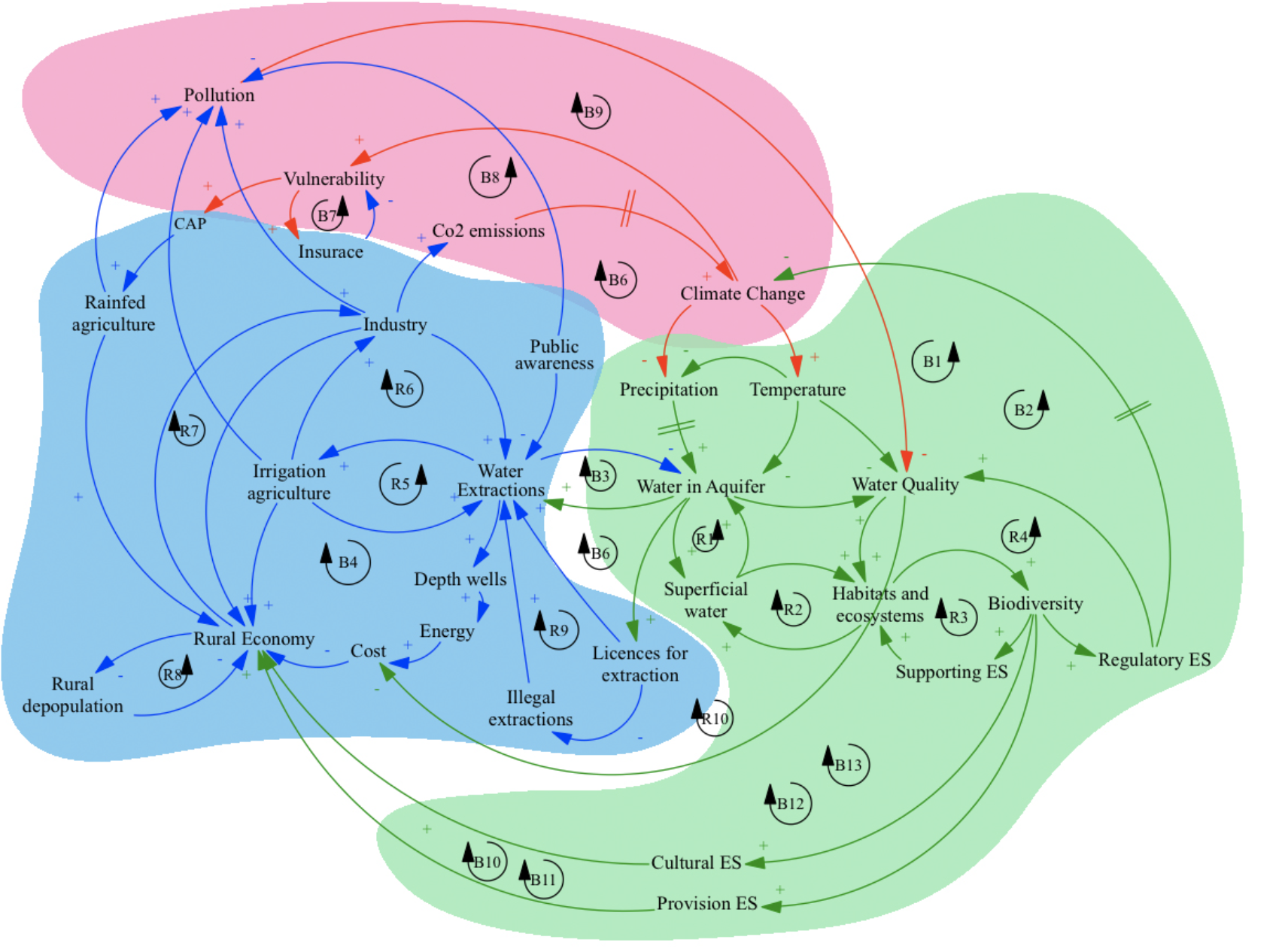


Figure A1. Qualitative System Dynamics Model co-developed with stakeholders. Highlighted in green the environmental variables and relationships, in blue the socio-economic elements are represented and in red the risks. The polarity of the relationship is represented by a positive (+) or negative (-) symbol. The processes or decisions that require some time to occur are indicated with a delay mark (//). Relevant feedback loops are numbered and their polarity and direction have been indicated: Reinforcing or positive loop (R), Balancing or negative loop (B).
