# Supplementary material for: Assessing the long-term effectiveness of Nature-Based Solutions under different climate change scenarios
Source: Sci Total Environ. 2021 Nov 10;794:148515. doi: 10.1016/j.scitotenv.2021.148515 (PMC8434435; doi:10.1016/j.scitotenv.2021.148515)
Supplement: Appendix B — Table B.1 List of equations and parameter values. [file mmc2.docx]

**Appendix B**

*Table B.1. List of equations and parameter values*

| **Variable Name** | **Variable Type and Sub-type** | **Units** | **Variable Description** |
| --- | --- | --- | --- |
| Basic soil infiltration (fc) | Constant-Normal | mm/day | *The value of fc corresponds to the permeability of the saturated soil in the first 30 centimetres of depth, considering that this is the soil layer that is in direct contact with rainwater- characteristic values of each type of soil.*  **Value:** High permeability (i.e. Sand) 1200; Medium Permeability (i.e. Sandy Loam) 600 ***^1^**; Low permeability (i.e. Clay) 12 |
|  |  |  | - Presented in View 1 - Used by “Soil texture (Kfc)”   **Reference: [2], [6], [13]** |
| Vegetation fraction (Kv) | Constant-Normal | Dmnl | *Fraction of rain that infiltrates due to vegetation cover.*  **Value:** Pastures <50%, Kv = 0.09; Cultivated land, Kv = 0.10 ***^1^**; Grassland coverage, Kv = 0.18; Forests, Kv=0.20;  Pasture coverage more than 75% = 0.21   - Presented in View 1 |
|  |  |  | - Used by “Infiltration coefficient”   **Reference: [13]** |
|  |  |  |  |
| Slope (Kp) | Constant-Normal | Dmnl | *Infiltration fraction due to slope effect.*  **Value:** Very flat (0.02% - 0.06%), Kp = 0.3; Flat (0.3% -0.4%), Kp = 0.2; Flat (1% - 2%), Kp = 0.15; Average (2% -7%), Kp = 0.10 ***^1^**; Strong (> 7%), Kp = 0.06 |
|  |  |  | - Presented in View 1 |
|  |  |  | - Used by “Infiltration coefficient”   **Reference: [4], [13]** |
| Soil texture (Kfc) | Auxiliary-Normal | Dmnl | *Fraction that infiltrates due to soil texture. To apply this equation, the range of basic soil infiltration (fc) has to be between 16 to 1568 mm / day. For values of fc less than 16 mm / day, Kfc = 0.0148fc / 16. For values of fc greater than 1568 mm / day, Kfc = 1.*  **Equation:**  IF THEN ELSE ("basic soil infiltration (fc)">=16: AND: "basic soil infiltration (fc)" <=1568, (0.267*LN (“basic soil infiltration (fc)”)) -(0.000154*"basic soil infiltration (fc)")-0.723, IF THEN ELSE (“basic soil infiltration (fc)"<16, (0.0148*"basic soil infiltration (fc)")/16, 1)) |
|  |  |  | - Presented in View 1 |
|  |  |  | - Used by “Infiltration coefficient”   **Reference: [2], [4], [6], [9], [13]** |
| Infiltration coefficient | Auxiliary-Normal | Dmnl | *The infiltration coefficient is the factor used to calculate the water that is infiltrated monthly to the ground. The infiltration is higher in areas with low slope and high vegetative cover.*  **Equation**  Infiltration coefficient= IF THEN ELSE (("Slope (Kp)"+"Soil texture (Kfc)"+"Vegetation fraction (Kv)")>1, 1, "Slope (Kp)"+"Soil texture (Kfc)"+"Vegetation fraction (Kv)”) |
|  |  |  | - Presented in View 1 |
|  |  |  | - Used by” Infiltration”   **Reference: [13]** |
| RCP2.6 | Data-Equation | mm/month | *Representative Concentration Pathway (RCP) is a greenhouse gas concentration trajectory adopted by the IPCC. The RCP2.6 mitigation scenarios aiming to limit the increase of global mean temperature to 2°C. RCP 2.6 requires that carbon dioxide (CO2) emissions start declining by 2020 and go to zero by 2100.* |
|  |  |  | **Equation:**  "RCP2.6”: = GET XLS DATA (‘? SDclima’, 'RCP2.6’, 'P’, 'R3’) |
|  |  |  | - Presented in View 1 |
|  |  |  | - Used by “precipitation”   **See Appendix C from supplementary material** |
| Rcp4.5 | Data-Equation | mm/ month | *Intermediate stabilisation pathways in which radiative forcing is stabilised at approximately 4.5 W m^-2^ and 6.0 W m^-2^ after 2100.* |
|  |  |  | **Equation:**  RCP4.5”: INTERPOLATE: = GET XLS DATA (‘? SDclima’, 'RCP4.5’, 'K', 'D3') |
|  |  |  | - Presented in View 1 |
|  |  |  | - Used by “precipitation”   **See Appendix C from supplementary material** |
| RCP8.5 | Data-Equation | mm/ month | *One high pathway for which radiative forcing reaches greater than 8.5 W m^-2^ by 2100 and continues to rise for some amount of time (the corresponding ECP assuming constant emissions after 2100 and constant concentrations after 2250).* |
|  |  |  | **Equation:** "RCP8.5”: INTERPOLATE: = GET XLS DATA (‘?SDclima' , 'RCP8.5' , 'L' , 'D3' ) |
|  |  |  | - Presented in View 1 |
|  |  |  | - Used by “precipitation”   **See Appendix C from supplementary material** |
| Precipitation | Data-Equation | mm/ month | *Projected precipitation in Medina del Campo under three different climate change scenarios* |
|  |  |  | **Equation:**  Precipitation: INTERPOLATE: = ("RCP2.6"*0) +("RCP4.5"*0) +("RCP8.5"*1) |
|  |  |  | - Presented in View 1 |
|  |  |  | - Used by “infiltration”, “interception” and “runoff” |
| Interception | Auxiliary-Normal | mm/ month | *Monthly precipitation intercepted by foliage.* |
|  |  |  | **Equation:**  Interception= IF THEN ELSE (Precipitation<=5, Precipitation, IF THEN ELSE (Precipitation>=5: AND: (Foliage coefficient*Precipitation)>=5, Foliage coefficient *Precipitation, 5 )) |
|  |  |  | - Presented in View 1 |
|  |  |  | - Used by “Infiltration” and “runoff”   **Reference: [13]** |
| Foliage coefficient | Constant-Normal | Dmnl | *Percentage of monthly rainfall that is retained in the foliage.*  **Value:** 0.12 in cultivated area |
|  |  |  | - Presented in View 1 |
|  |  |  | - Used by “interception”.   **Reference: [13]** |
| Runoff | Auxiliary-Normal | mm/  month | *Flow of water occurring on the ground surface when excess rainwater is not sufficiently infiltrated into the soil.* |
|  |  |  | **Equation:**  runoff= MAX (Precipitation-infiltration-Interception, 0) |
|  |  |  | - Presented in View 1 |
|  |  |  | - Used by “Inflow water Adaja”, “Inflow water Guareña”, “Inflow water Trabancos”, “Inflow water Zapardiel”, “Inflow water Wetland”   **Reference: [13], [19]** |
| Infiltration | Auxiliary-Normal | mm/  month | *Monthly precipitation that enters into the soil.* |
|  |  |  | **Equation:**  infiltration= (Infiltration coefficient*((Precipitation + Artificial recharge)-Interception)) + ((Infiltration coefficient *((Precipitation + Artificial recharge)-Interception) *Soil conservation practices)) |
|  |  |  | - Presented in View 1 |
|  |  |  | - Used by “Soil moisture”, “Percolation”, “Runoff” and “water excess”   **Reference: [2], [4], [5],[8],[13]** |
| Initial soil moisture | Constant-Normal | mm | *Soil moisture at Time step 0* |
|  |  |  | **Value:** 50 |
|  |  |  | - Presented in View 1 |
|  |  |  | - Used by “soil moisture”   **Reference: [2], [4], [5]** |
| Soil moisture | Level-Normal | mm | *Amount of water stored in the soil* |
|  |  |  | **Equation:**  Soil moisture= INTEG (infiltration-Evaporation-Percolation, initial soil moisture) |
|  |  |  | - Presented in View 1 |
|  |  |  | - Used by “evaporation” and “saturation”. |
| Percolation | Auxiliary-Normal | mm/  month | *Water that moves from soil storage to groundwater per month.* |
|  |  |  | **Equation:**  Percolation= water excess+(POWER((saturation/100), exponent)) *infiltration*percolation factor |
|  |  |  | - Presented in View 1 |
|  |  |  | - Used by “total recharge”   **Reference: [13], [19]** |
| Percolation factor | Constant-Normal | Dmnl | **Value**: 0.03 |
|  |  |  | - Hidden variable |
|  |  |  | - Used by “percolation” |
| Exponent | Constant-Normal | Dmnl | **Value:** 2 |
|  |  |  | - Hidden variable |
|  |  |  | - Used by “percolation” |
| Evaporation | Auxiliary-Normal | mm/ month | *Quantity of soil water that is transpired and retained in plant tissues, and evaporated from surrounding soil surfaces. It is assumed that when soil moisture is lower than the wilting point, the plants close their stoma. Consequently, the evapotranspiration stops.* |
|  |  |  | **Equation:** Evaporation= IF THEN ELSE (Soil moisture>Wilting point, ETP, 0) |
|  |  |  | - Presented in view 1 |
|  |  |  | - Used by “soil moisture”, “Percolation”, “Runoff” and “water excess” |
| ETP | Data-Equation | mm/  month | *Potential evapotranspiration under three different climate change scenarios.* |
|  |  |  | **Equation:**  ETP: INTERPOLATE: = ("RCP 2.6"*0) +("RCP 4.5"*0) +("RCP 8.5"*1) |
|  |  |  | The ETP was calculated in Excel and integrated in the SD model using GET EXL DATA function. The following equation was used: ETP=(8.10+0.46*T)*Ps where, T= Mean monthly temperature in C^0^.  P= Percentage of monthly sunlight with respect to the year [%]. |
|  |  |  | - Presented in View 1 |
|  |  |  | - Used by “evapotranspiration” |
| RCP 2.6 | Data-Equation | mm/ month | *Evapotranspiration calculated using temperature in scenario RCP 2.6* |
|  |  |  | **Equation:**  RCP 2.6: = GET XLS DATA (‘?SDclima' , 'RCP2.6' , 'P' , 'Q3' ) |
|  |  |  | - Presented in View 1 |
|  |  |  | - Used by “ETP”   **See Appendix C from supplementary material** |
| RCP 4.5 | Data-Equation | mm/  month | *Evapotranspiration calculated using temperature in scenario RCP 4.5* |
|  |  |  | **Equation:**  "RCP 4.5”: = GET XLS DATA (‘?SDclima' , 'RCP4.5' , 'K' , 'M3' ) |
|  |  |  | - Presented in View 1 |
|  |  |  | - Used by “ETP”   **See Appendix C from supplementary material** |
| RCP 8.5 | Data-Equation | mm/  month | *Evapotranspiration calculated using temperature in scenario RCP 8.5* |
|  |  |  | **Equation:**  "RCP 8.5”: = GET XLS DATA (‘?SDclima' , 'RCP8.5' , 'L' , 'N3' ) |
|  |  |  | - Presented in View 1 |
|  |  |  | - Used by” ETP”   **See Appendix C from supplementary material** |
| Wilting point | Constant-Normal | mm | *Point of minimum humidity at which a plant cannot continue to extract water from the soil and cannot recover from water loss even if the ambient humidity is saturated. In the model an estimation of the wilting point was used (17% of the root zone).* **Value used in the model:** Wilting point: 103 mm |
|  |  |  | - Presented in view 1 |
|  |  |  | - Used by “Evapotranspiration”   **Reference: [4], [13]** |
| Saturation | Auxiliary-Normal | Dmnl | *Amount of soil pores that are filled with water. The soil is saturated when it reaches the field capacity. When the soil is saturated the percolation is higher.* |
|  |  |  | **Equation:**  saturation= (Soil moisture/field capacity) *100 |
|  |  |  | - Presented in View 1 |
|  |  |  | - Used by “percolation” and “water excess” |
| Field Capacity | Constant-Normal | mm | *Soil moisture condition at which the soil contains the maximum amount of water that can hold before it percolates. In other words, it is the maximum water storage in soil. It has been estimated that the Field capacity in Medina del Campo corresponds to the 35% of the root zone.* |
|  |  |  | **Value used in the model:** 210mm |
|  |  |  | - Presented in View 1 |
|  |  |  | - Used by “saturation” and “water excess”   **Reference: [4]** |
| Water excess | Auxiliary-Normal | mm/  month | *Difference between the maximum amount of water in the soil (on its saturated point) and the water that percolates.* |
|  |  |  | **Equation:** water excess=MAX(((saturation/100) *(field capacity/TIME STEP) +infiltration-(field capacity/TIME STEP)), 0) |
|  |  |  | - Presented in View 1 |
|  |  |  | - Used by “percolation” |
| Percentage of infiltration increase | Constant-Normal | Dmnl | *Percentage of infiltration increase due to soil conservation practices. Techniques to improve soil quality by preventing soil degradation and building organic matter. These practices include crop rotation, tillage reduction, mulching or cover cropping. A number of studies have estimated that conservation practices may increase soil infiltration up to 30%.* |
|  |  |  | **Value in the model**: 0.20 |
|  |  |  | - Presented in View 1 |
|  |  |  | - Used by “infiltration” and “regeneration”   **Reference: [1], [3], [14], [16], [17]** |
| Irrigation return | Auxiliary-Normal | mm/  month | *Fraction of irrigation water that returns to the system.* |
|  |  |  | **Equation:** Irrigation return= (((Irrigation demand*conversion factor*Factor mm)/river basin area) *Percentage of irrigation returning)/TIME STEP |
|  |  |  | - Presented in View 1 |
|  |  |  | - Used by “infiltration”   **Reference: [7], [9]** |
| Percentage of irrigation returning | Constant-Normal | Dmnl | *Percentage of water that returns into the system. It is assumed that this percentage reduces when efficient irrigation systems are implemented. It has been estimated that 35% of the water used by agriculture returns into the system.*  **Value:** 0.35 |
|  |  |  | - Presented in View 1 |
|  |  |  | - Used by irrigation return   **References: [7], [9], [15]** |
| Irrigation demand | Auxiliary-Normal | Hm*Hm*Hm | *Water used by irrigation crops in the area of Medina del Campo* |
|  |  |  | **Equation:** Irrigation demand=Cereals water demand + Forage crops water demand + Industrial crops water demand +Other irrigation crops WC + Vegetables water demand |
|  |  |  | - Presented in View 4 |
|  |  |  | - Used by “GW extractions” and “irrigation demand” |
| River Basin Area | Constant-Normal | m*m | *Area of Medina del Campo Groundwater body* |
|  |  |  | **Value:** 3700000000 m2 |
|  |  |  | - Presented in View 1 |
|  |  |  | - Used by “Cereals area”, “Forage crops area”, “Industrial crops area”, “Vegetable crops area”, “Other crops area”, “Piezometric level”, “Irrigation return”, “Groundwater” |
| Factor mm | Constant-Normal | mm | *Factor to covert units to convert (m*m*/m*m) into mm* |
|  |  |  | **Value: 1** |
|  |  |  | Hidden variable |
| Conversion factor | Constant-Normal | m*m/  (Hm*Hm*Hm) | *Factor to convert to Hm3 into litters* |
|  |  |  | **Value:** 1000000 |
|  |  |  | Hidden variable |
| Artificial recharge NBS | Constant-Normal | Hm*Hm*Hm | *The recharge will take place by means of a surface technique, by pouring into the channel (in Trabancos and Zapardiel river).* |
|  |  |  | **Value** of monthly recharge volume in Zapardiel: 0.77 |
|  |  |  | - Presented in view 2 |
|  |  |  | - Used by "inflow water in Zapardiel"   **Reference: [4], [19]** |
| Artificial recharge NBS2 | Constant-Normal | Hm*Hm*Hm | *The recharge will take place by means of a surface technique, by pouring into the channel (in Trabancos and Zapardiel river).* |
|  |  |  | **Value** of monthly recharge volume in Trabancos: 0.6 |
|  |  |  | - Presented in view 2 |
|  |  |  | - Used by “Inflow water in Trabancos”   **Reference: [4], [19]** |
| Total recharge | Auxiliary-Normal | Hm*Hm*Hm/  month | *Amount of water that recharges the aquifer.* |
|  |  |  | **Equation:** TOTAL RECHARGE= (factor*Percolation*Permeable surface) +groundwater lateral movement + Zapardiel drainage +Trabancos |
|  |  |  | - Presented in view 1 |
|  |  |  | - Used by “groundwater” |
| factor | Constant-Normal | (Hm*Hm*Hm)/  (m*m* mm) | *Units converting factor (From mm/month to hm3). Precipitation is in mm/month. This means that each mm is one litre per m2. For example, if it rains 380 mm/month. This means that each month it rains 380 litres of water per m2.* |
|  |  |  | **Value:** 1e-09 |
| groundwater lateral movement | Constant-Normal | Hm*Hm*Hm/ month | *Water coming from other GW bodies.* |
|  |  |  | **Value**: 0.75 |
|  |  |  | - Presented in View 1 |
|  |  |  | - Used by “total recharge”   **Reference: [5],[6]** |
| Permeable surface | Auxiliary-Normal | m*m | *Area of basin that is permeable*  **Value**: 3184*1e+06   - Presented in view 1 - Used by “TOTAL RECHARGE”   **Reference: [5],[6]** |
| Trabancos drainage | auxiliary-Normal | Hm*Hm*Hm | *Amount of water that drains into the aquifer from Trabancos river*  **Equation:**  IF THEN ELSE (Trabancos river<=0, 0, 0.0275*Trabancos river) |
|  |  |  | - Presented in View 1 |
|  |  |  | - Used by “total recharge”   **Reference: [8], [6], [12]** |
| Zapardiel drainage | auxiliary-Normal | Hm*Hm*Hm | *Amount of water that drains into the aquifer from Zapardiel river.* |
|  |  |  | **Equation:**  Zapardiel drainage= IF THEN ELSE (Zapardiel river<=0, 0, 0.112) |
|  |  |  | - Presented in View 2 |
|  |  |  | - Used by "outflow Trabancos" and "Total recharge"   **Reference: [8], [6], [12]** |
| groundwater inflow | auxiliary-Normal | Hm*Hm*Hm/  month | *Amount of water that goes into the aquifer, corresponds to total recharge.* |
|  |  |  | - Presented in View 1 |
|  |  |  | - Used by “groundwater” |
| Groundwater | Level-Normal | Hm*Hm*Hm | *Amount of water in the aquifer at time t.* |
|  |  |  | **Equation:**  groundwater= INTEG (groundwater inflow-groundwater outflow, river basin area*factor de conversion m3 a Hm3*Initial piezometric level) |
|  |  |  | - Presented in View 1 |
|  |  |  | - Used by “Piezometric level” |
| Groundwater outflow | auxiliary-Normal | Hm*Hm*Hm/  month | *Water that goes out of the aquifer.* |
|  |  |  | **Equation:**  groundwater outflow= (discharge to superficial ecosystems + GW extractions + illegal extractions)/TIME STEP |
|  |  |  | - Presented in view 1 |
|  |  |  | - Used by “groundwater” |
| GW extractions | auxiliary-Normal | Hm*Hm*Hm | *Amount of water extracted from the aquifer.* |
|  |  |  | **Equation:** GW extractions=MIN (Urban water demand + Irrigation demand, Maximum volume of water allowed for extractions) |
|  |  |  | - Presented in view 1 |
|  |  |  | - Used by “groundwater outflow” |
| Urban water demand | constant-Normal | Hm*Hm*Hm | *Urban* water demand per month. |
|  |  |  | **Value:** 0.27 |
|  |  |  | - Presented in View 1 |
|  |  |  | - Used by “GW extractions”   **Reference: [4], [5], [9]** |
| Maximum volume of water allowed for extractions | constant-Normal | Hm*Hm*Hm | *Maximum volume of water that the river basin authority allows extracting. It is assumed that the maximum volume of water allowed for extractions equals to the current demand for water.* |
|  |  |  | **Value:** In BAU scenario: 21.7; In scenario with GW extractions restrictions: 17.3 |
|  |  |  | - Presented in View 1 |
|  |  |  | - Used by “GW extractions”   **Reference: [4], [5], [9]** |
| Illegal extractions | Auxiliary with Lookup | Hm*Hm*Hm | *Volume of water extracted from the aquifer without permission. The model assumes that illegal extractions reduce when the environmental awareness increase.* |
|  |  |  | **Equation:**  illegal extractions= WITH LOOKUP (Environmental awareness, ([(0,0(10,10)], (0,1), (0.2,0.8), (0.3,0.7), (0.4,0.5), (0.5,0.12), (0.6,0.15), (0.7,0.1), (0.8,0.08), (0.9,0.05), (1,0) )) |
|  |  |  | - Presented in View 1 |
|  |  |  | - Used by “GW extractions” |
| Environmental awareness | Auxiliary-Normal | Dmnl | *The level at which the rural population and tourists are aware of the impacts that their behaviour impacts the environment and the level of commitments to make changes.* |
|  |  |  | **Equation:**  DELAY1I ((3*"Level of information/Citizens engagement”) +profit, 24, 0.1) |
|  |  |  | - Presented in view 3 |
|  |  |  | - Used by "environmental stress" and "illegal extractions" |
| Discharge to superficial ecosystems | Auxiliary-Normal | Hm*Hm*Hm | *Amount of water that is discharged to superficial ecosystems. The discharge happens when the piezometric level is height enough to reach the basin of superficial ecosystems.* |
|  |  |  | **Equation:**  discharge to superficial ecosystems= GW discharge to Adaja + GW discharge to Guareña + GW discharge to trabancos + GW discharge to Zapardiel + GW discharge to wetland |
|  |  |  | - Presented in View 1 |
|  |  |  | - Used by” groundwater outflow” |
| GW discharge to Adaja | Auxiliary-Normal | Hm*Hm*Hm | *Amount of groundwater that is discharged to Adaja river.* |
|  |  |  | **Equation:**  GW discharge to Adaja =IF THEN ELSE (Piezometric level>Height Adaja, (Piezometric level-Height Adaja). *Area of water in Adaja*factor de conversion m3 a Hm3, 0) |
|  |  |  | - Presented in view 2 |
|  |  |  | - Used by "discharge to superficial ecosystems" and "Inflow Adaja" |
| GW discharge to Guareña | Auxiliary-Normal | Hm*Hm*Hm | *Amount of groundwater that is discharged to Guareña river.* |
|  |  |  | **Equation:**  GW discharge to Guareña =IF THEN ELSE (Piezometric level>Height Guareña , (Piezometric level-Height Guareña)*Area of water in Guareña*factor de conversion m3 a Hm3 , 0 ) |
|  |  |  | - Presented in view 2 |
|  |  |  | - Used by "discharge to superficial ecosystems" and "Inflow Guareña" |
| GW discharge to Trabancos | Auxiliary-Normal | Hm*Hm*Hm | *Amount of groundwater that is discharged to Trabancos river.* |
|  |  |  | **Equation:**  GW discharge to Trabancos =IF THEN ELSE (Piezometric level>Height Trabancos, (Piezometric level-Height Trabancos) *Area of water in Trabancos*factor de conversion m3 a Hm3, 0) |
|  |  |  | - Presented in view 2 |
|  |  |  | - Used by "discharge to superficial ecosystems" and "Inflow Trabancos" |
| GW discharge to Zapardiel | Auxiliary-Normal | Hm*Hm*Hm | *Amount of groundwater that is discharged to Zapardiel river.* |
|  |  |  | **Equation:**  GW discharge to Zapardiel =IF THEN ELSE (Piezometric level>Height Zapardiel, (Piezometric level-Height Zapardiel) *Area of water in Zapardiel*factor de conversion m3 a Hm3, 0) |
|  |  |  | - Presented in view 2 |
|  |  |  | - Used by "discharge to superficial ecosystems" and "Inflow Zapardiel |
| GW discharge to wetland | Auxiliary-Normal | Hm*Hm*Hm | *Amount of groundwater that is discharged to the wetlands.* |
|  |  |  | **Equation:**  GW discharte to wetland=IF THEN ELSE (Piezometric level>=Discharge treshold level, ((Piezometric level -Discharge treshold level) *Wetlands area*factor de conversion m3 a Hm3), 0) |
|  |  |  | - Presented in view 2 |
|  |  |  | - Used by "discharge to superficial ecosystems" and "Inflow Wetland" |
| Piezometric level | Auxiliary-Normal | m | *Water levels of the aquifer’s basin* |
|  |  |  | **Equation:**  Piezometric level= (groundwater*units’ factor)/river basin area |
|  |  |  | - Presented in view 1 |
|  |  |  | - Used by "Depth of wells", " GW dischafge to Adaja", "GW discharge to Guareña","GW discharge to trabancos", "GW discharge to Zapardiel" and "GW discharge to wetland" |
| Initial piezometric level | Constant-Normal | m | *Groundwater level at the beginning of the simulation. Value measured for height 730m.* |
|  |  |  | **Value**: 665 |
|  |  |  | - Presented in View 1 |
|  |  |  | - Used by “groundwater”   **Reference: [4], [5], [6]** |
| depth of wells | Auxiliary-Normal | m | *Depth of wells used to extract groundwater.* |
|  |  |  | **Equation:**  depth of wells=area height-Piezometric level |
|  |  |  | - Presented in View 1 |
|  |  |  | - Used by “cost of extracting water” |
| area height | constant-Normal | m | *Height of the Basin (Metres Above Sea Level)* |
|  |  |  | **Value**: 740 |
|  |  |  | - Presented in view 1 |
|  |  |  | - Used by "depth of wells"   **Reference: [4], [6]** |
| Height Adaja | Constant-Normal | m | *Source of river at 1490m, Mouth of the river at 680m* |
|  |  |  | **Value:** For the simulation a height of 800m has been chosen |
|  |  |  | - Presented in view 2 |
|  |  |  | - Used by “GW discharge to Adaja”   **Reference: [12], [9]** |
| Area of water in Adaja | constant-Normal | m*m | *River basin area* |
|  |  |  | **Value:** 5.304e+09 |
|  |  |  | - Presented in view 2 |
|  |  |  | - Used by "Inflow water Adaja" and "Outflow water Adaja"   **Reference: [12], [9]** |
| Adaja river | Level-Normal | Hm*Hm*Hm | *Amount of water in Adaja river at time t. Initial value: 34.375 (Average flow in a month)* |
|  |  |  | **Equation:** Adaja river = INTEG (Inflow water Adaja-outflow Adaja, 34.375) |
|  |  |  | - Presented in view 2 |
|  |  |  | - Used by "Extractions Adaja", "Outflow Adaja" and "Quality Adaja" |
| Ecological flow from Cogotas reservoir | constant-Normal | Hm*Hm*Hm | *The River Basin Authority (Confederación hidrological del Duero) has allowed an ecological flow from the Cogotas dam to the Adaja river of 13.08 cubic hectometres per year in normal periods and 8.23 hm3 in periods of drought. The ecological flow varies from month to month. The model assumes that the ecological flow from the Cogotas dam is constant over the year.* |
|  |  |  | **Value:** 0.9 |
|  |  |  | - Presented in View 2 |
|  |  |  | - Used by "Inflow water in Adaja" and "Extractions Adaja"   **Reference: [12], [7], [8]** |
| Outflow Adaja | auxiliary-Normal | Hm*Hm*Hm  /month | *Water that goes out of the Adaja river. The model assumes that the only variables affecting the outflow are Evapotranspiration and superficial extractions. It does not consider the flow to other rivers or streams.* |
|  |  |  | **Equation:**  MIN(((Area of water in Adaja*ETP*factor) + ( extractions Adaja/TIME STEP)),Adaja river/TIMESTEP) |
|  |  |  | - Presented in View 2 |
|  |  |  | - Used by “Adaja river” |
| Extractions Adaja | auxiliary-Normal | Hm*Hm*Hm | *Water extracted for irrigation. The model assumes that an ecological flow is maintained over the year. For this reason, extractions from Adaja will be produced if there is enough water to maintain this ecological flow*. |
|  |  |  | **Equation:**  extractions Adaja=IF THEN ELSE (Adaja river <= Superficial water extractions, 0, Adaja river -Ecological flow from Cogotas reservoir) |
|  |  |  | - Presented in view 2 |
|  |  |  | - Used by "Outflow Adaja" |
| Superficial water extractions | constant-Normal | Hm*Hm*Hm | *Monthly water extractions from Adaja river* |
|  |  |  | **Value:** 3.54 |
|  |  |  | - Presented in View 2 |
|  |  |  | - Used by "Extractions Adaja"   **Reference: [10]** |
| Height Zapardiel | Constant-Normal | m | *Source of river at 1150m, Mouth of the river at 675m* |
|  |  |  | **Value:** For the simulation a height of 700m has been chosen |
|  |  |  | - Presented in view 2 |
|  |  |  | - Used by “GW discharge to Zapardiel”   **Reference: [12], [9]** |
| Inflow water on Zapardiel | auxiliary-Normal | Hm*Hm*Hm/  month | *Water that flows into Zapardiel river. Water from runoff or artificial recharge NBS (If the variable is activated).* |
|  |  |  | **Equation:**  Inflow water on Zapardiel=MAX (((Area of water in Zapardiel*factor*runoff) +(((Artificial recharge NBS)+GW discharge to Zapardiel-deviation to channels)/TIME STEP)) , 0 ) |
|  |  |  | - Presented in view 2 |
|  |  |  | - Used by "Zapardiel river" |
| Deviation channels | constant-Normal | Hm*Hm*Hm | *Amount of water that is deviated through channels. There is a network of canalizations and diversions that extract the waters of torrential episodes.* |
|  |  |  | **Value:** 5.2 |
|  |  |  | - Presented in view 2 |
|  |  |  | - Used by "Inflow water on Zapardiel"   **Reference: [8]** |
| outflow Zapardiel | auxiliary-Normal | Hm*Hm*Hm/  month | *Water that goes out of the Zapardiel river. The model only considers Evapotranspiration and Zapardiel drainage. It does not consider the flow to other rivers or streams.* |
|  |  |  | **Equation:**  outflow Zapardiel= MIN ((factor*ETP*Area of water in Zapardiel+(Zapardiel drainage/TIME STEP)) , Zapardiel river/TIME STEP ) |
|  |  |  | - Presented in View 2 |
|  |  |  | - Used by Zapardiel river |
| Height Guareña | Constant-Normal | m | *Source of river at 932m, Mouth of the river at 700m* |
|  |  |  | **Value:** For the simulation a height of 720m has been chosen |
|  |  |  | - Presented in view 2 |
|  |  |  | - Used by “GW discharge to Guareña”   **Reference: [12]** |
| Area of Water in Guareña | constant-Normal | m*m | River basin area |
|  |  |  | **Value:** 1.007e+09 |
|  |  |  | Presented in view 2 |
|  |  |  | Used by "Inflow water Guareña" and "Outflow water Guareña"  **Reference: [12]** |
| Inflow water on Guareña | auxiliary-Normal | Hm*Hm*Hm/  month | *Water that flows into Guareña river. Water from runoff and GW discarge to Guareña.* |
|  |  |  | **Equation:**  Inflow water on Guareña= (Area of water in Guareña*factor*runoff) +((GW discharge to Guareña)/TIME STEP) |
|  |  |  | - Presented in view 2 |
|  |  |  | - Used by "Guareña river" |
| Guareña river | Level-Normal | Hm*Hm*Hm | *Amount of water in Guareña river at time t. Initial value: 5.3 (Average flow in a month)* |
|  |  |  | **Equation:** Guareña river = INTEG (Inflow water on Guareña-Outflow Guareña, 5.3) |
|  |  |  | - Presented in view 2 |
|  |  |  | - Used by “Guareña drainage”, “Outflow Guareña” and “Quality Guareña”   **Reference: [12]** |
| Outflow Guareña | auxiliary-Normal | Hm*Hm*Hm/  month | *Water that goes out of the Guareña river. The model assumes that the only variables affecting the outflow are Evapotranspiration and Guareña drainage. It does not consider the flow to other rivers or streams.* |
|  |  |  | **Equation:**  Outflow Guareña=MIN (((factor*ETP*Area of water in Guareña+(Gruareña drainage/TIME STEP))) , (Guareña river/TIME STEP) ) |
|  |  |  | - Presented in view 2 |
|  |  |  | - Used by “Guareña river” |
| Guareña drainage | auxiliary-Normal | Hm*Hm*Hm | *Amount of water that drains from Guareña river.* |
|  |  |  | **Equation:**  Gruareña drainage=IF THEN ELSE (Guareña river<=0, 0 , 0.1255*Guareña river ) |
|  |  |  | - Presented in view 2 |
|  |  |  | - Used by "Gruareña outflow" |
| Height Trabancos | Constant-Normal | m | *Source of river at 1120m, Mouth of the river at 657m* |
|  |  |  | **Value:** For the simulation a height of 720m has been chosen |
|  |  |  | - Presented in view 2 |
|  |  |  | - Used by “GW discharge to Trabancos”   **Reference: [12]** |
| Inflow water on Trabancos | auxiliary-Normal | Hm*Hm*Hm/  month | *Water that flows into Trabancos river. Coming from runoff, GW discharge to Trabancos or artificial recharge NBS (If the variable is activated).* |
|  |  |  | **Equation:**  Inflow water on Trabancos=MAX (((Area of water in Trabancos*factor*runoff) + (((Artificial recharge NBS2) +GW discharge to Trabancos)/TIME STEP)), 0) |
|  |  |  | - Presented in view 2 |
|  |  |  | - Used by "Zapardiel river" |
| Trabancos river | Level-Normal | Hm*Hm*Hm | *Amount of water in Trabancos river at time t. Initial value: 6.425 (Average flow in a month).* |
|  |  |  | **Equation:**  Trabancos river = INTEG (inflow water on Trabancos-outflow Trabancos, 6.425) |
|  |  |  | - Presented in view 2 |
|  |  |  | - Used by "Trabancos river" |
| Outflow Trabancos | auxiliary-Normal | Hm*Hm*Hm/  month | *Water that goes out of the Trabancos river. The model assumes that the only variables affecting river outflow are Evapotranspiration and Trabancos drainage. It does not consider the flow to other rivers or streams.* |
|  |  |  | **Equation:**  outflow Trabancos= MIN ((factor*ETP*Area of water in Trabancos+ (Trabancos drainage/TIME STEP)), Trabancos river/TIME STEP) |
|  |  |  | - Presented in View 2 |
|  |  |  | - Used by “Trabancos river” |
| Discharge threshold level | Constant-Normal | m | *Height at which the aquifer discharges to the wetland.* |
|  |  |  | **Value:** 720m |
|  |  |  | - Presented in view 2 |
|  |  |  | - Used by “GW discharge to Wetland”   **Reference: [12],[4],[18]** |
| Inflow water on wetland | auxiliary-Normal | Hm*Hm*Hm/  month | *Water that flows into Wetland. Water from runoff and GW discharge to Wetland.* |
|  |  |  | **Equation:**  inflow water on wetland=(GW discharge to wetland/TIME STEP)+(factor*Precipitation*Wetlands area)+(factor*Wetlands area*runoff) |
|  |  |  | - Presented in view 2 |
|  |  |  | - Used by "Wetland" |
| Wetland | Level-Normal | Hm*Hm*Hm | *Amount of water in Wetland at time t. Initial value: 0.122* |
|  |  |  | **Equation:**  Wetland= INTEG (inflow water on wetland-wetland water outflow, 0.122) |
|  |  |  | - Presented in View 1 |
|  |  |  | - Used by "Quality Wetland", "Wetland drainage" and "wetland water outflow" |
| Wetland water outflow | auxiliary-Normal | Hm*Hm*Hm/  month | *Amount of water that is lost due to Evapotranspiration or drainage to GW aquifer.* |
|  |  |  | **Equation:**  wetland water outflow=MIN ((ETP*Wetlands area*factor) + (wetland drainage/TIME STEP) , (Wetland/TIME STEP)) |
|  |  |  | - Presented in view 1 |
|  |  |  | - Used by "Wetland" |
| wetland drainage | auxiliary-Normal | Hm*Hm*Hm | *Percentage of wetland water that drains into the aquifer.* |
|  |  |  | **Equation:**  wetland drainage =IF THEN ELSE (Wetland<=0 , 0 , permeability of soil*Wetland ) |
|  |  |  | - Presented in View 1 |
|  |  |  | - Used by "Wetland outflow" |
| Wetland area | constant-Normal | m*m | *Wetland area* |
|  |  |  | **Value:** 1.22e+06 |
|  |  |  | - Presented in view 2 |
|  |  |  | - Used by "Inflow water on Wetland" and "Outflow water on Wetland"   **Reference: [12],[4],[18]** |
| Quality Guareña | auxiliary-Normal | dmnl | *Quality status of Guareña river. The only criteria used to define the quality of the river is the amount of water in the river. A lookup table has been used. The model assumes that the higher the amount of water in the river the higher is the quality of the river.* |
|  |  |  | **Equation:**  Quality Guareña= WITH LOOKUP (Guareña river, ([(0,0)(100,10)],(0,0),(0.5,0),(1,0),(2,0.1),(2.5,0.15),(3,0.2),(3.5,0.25),(4,0.3),(4.5,0.4),(5,0.5),(5.5,0.6),(6,0.7),(7,0.8),(8,0.9),(9,1) )) |
|  |  |  | - Presented in view 3 |
|  |  |  | Used by "Quality of rivers" |
| Quality Adaja | auxiliary-Normal | dmnl | *Quality status of Adaja river. The only criteria used to define the quality of the river is the amount of water in the river. A lookup table has been used. The model assumes that the higher the amount of water in the river the higher is the quality of the river.* |
|  |  |  | **Equation:**  Quality Adaja= WITH LOOKUP (Adaja river,([(0,0)-(150,10)],(0,0),(6.8,0.1),(7.5,0.15),(9.5,0.2),(10.5,0.25),(11.5,0.3),(14,0.35),(16,0.4),(18,0.45),(20,0.5),(25,0.6),(30,0.7),(35,0.8),(40,1) )) |
|  |  |  | - Presented in view 3 |
|  |  |  | - Used by "Quality of rivers" |
| Quality Trabancos | auxiliary-Normal | dmnl | *Quality status of Trabanco river. The only criteria used to define the quality of the river is the amount of water in the river. A lookup table has been used. The model assumes that the higher the amount of water in the river the higher is the quality of the river.* |
|  |  |  | **Equation:**  Quality trabanco= WITH LOOKUP (Trabanco river, ([(0,0)(100,10)],(0,0),(1.28,0.1),(2,0.2),(3,0.3),(4,0.4),(5,0.45),(6,0.5),(7,0.6),(8,0.65),(9,0.7),(10,0.75),(11,0.8),(12,0.85),(13,1) )) |
|  |  |  | - Presented in view 3 |
|  |  |  | Used by "Quality of rivers" |
| Quality Zapardiel | auxiliary-Normal | dmnl | *Quality status of Zapardiel river. The only criteria used to define the quality of the river is the amount of water in the river. A lookup table has been used. The model assumes that the higher the amount of water in the river the higher is the quality of the river.* |
|  |  |  | **Equation:**  Quality Zapardiel= WITH LOOKUP (Zapardiel river, ([(0,0)(50,10)],(0,0),(0.8,0.1),(1,0.15),(1.5,0.2),(2,0.3),(2.5,0.4),(3,0.45),(4,0.5),(5,0.55),(6,0.6),(7,0.7),(8,0.8),(10,1) )) |
|  |  |  | - Presented in view 3 |
|  |  |  | Used by "Quality of rivers" |
| Quality river | auxiliary-Normal | dmnl | *Quality status of all rivers. The only criteria used to define the quality of the rivers is the amount of water in the river. A lookup table has been used. The model assumes that the higher the amount of water in the rivers the higher is the quality of the rivers.* |
|  |  |  | **Equation:** quality rivers= WITH LOOKUP ((Quality Adaja+Quality Guareña+Quality trabanco+Quality Zapardiel) *Units correction,([(0,0)(10,10)],(0,0),(0.6,0.1),(1,0.3),(2,0.5),(2.5,0.6),(3,0.7),(3.5,0.85),(4,1) )) |
|  |  |  | - Presented in view 3 |
|  |  |  | - Used by "Regeneration" |
| Quality wetland | auxiliary-Normal | dmnl | *Quality status of wetland. The only criteria used to define the quality of the wetlands is the amount of water in the wetland. A lookup table has been used. The model assumes that the higher the amount of water in the wetland the higher is the quality of the wetland.* |
|  |  |  | **Equation:** Quality wetland= WITH LOOKUP (Wetland*Units correction,([(0,0)(100,10)],(0,0),(0.05,0),(0.1,0),(0.15,0.2),(0.2,0.3),(0.25,0.5),(0.3,0.6),(0.35,0.7),(0.4,1),(4.45,1),(5,1),(100,1) )) |
|  |  |  | - Presented in view 3 |
|  |  |  | Used by "regeneration" |
| regeneration rate | constant-Normal | dmnl | *Rate at which ecosystems regenerates or recover their quality.* |
|  |  |  | **Value:** 0.05 |
|  |  |  | - Presented in view 3 |
|  |  |  | - Used by "regeneration" - Own elaboration. Based on Based on Bossel’s Systems Zoo |
| regeneration | auxiliary-Normal | dmnl/ month | *Ecosystems recovery per month. It is assumed that the ecosystems need some time to recover. Based on literature, we assume that they need 10 years.* |
|  |  |  | **Equation:**  regeneration= DELAY FIXED (((quality rivers + Quality wetland + Percentage of infiltration increase) *REGENERATION RATE) ,120, 0) |
|  |  |  | - Presented in view 3 |
|  |  |  | - Used by "Environmental quality" |
| Environmental quality | Level-Normal | dmnl | *Quality of Ecosystems at time t* |
|  |  |  | **Equation:**  Environmental quality= INTEG ((regeneration-degradation)/1, 0.5) |
|  |  |  | - Presented in view 3 |
|  |  |  | - Used by " degradation" and "attractiveness" |
| Degradation Rate | constant-Normal | dmnl | *Rate at which ecosystems degrade naturally.* |
|  |  |  | **Value:** 0.02 |
|  |  |  | - Presented in view 3 |
|  |  |  | - Used by "degradation" - Own elaboration. Based on Based on Bossel’s Systems Zoo |
| Degradation | auxiliary-Normal | dmnl/ month | *Degradation of environmental quality per month* |
|  |  |  | **Equation:**  degradation=DEGRADATION RATE*environmental stress*Environmental quality |
|  |  |  | - Presented in view 3 |
|  |  |  | - Used by "Environmental quality" |
| Advertise-ment | constant-Normal | dmnl | *Level of promotion to attract new tourists* |
|  |  |  | **Value:** 0-2 The simulations on this study have use a value of 2 |
|  |  |  | - Presented in view 3 |
|  |  |  | - Used by "Attractiveness"   Based on Based on Bossel’s Systems Zoo |
| attractiveness | auxiliary-Normal | dmnl | *Landscape appraisal level* |
|  |  |  | **Equation:**  attractiveness=advertisement*Environmental quality |
|  |  |  | - Presented in view 3 |
|  |  |  | Used by "tourist gain" |
| TOURIST RATE | constant-Normal | dmnl | *Rate at which tourist arrive to the area* |
|  |  |  | **Value:** 0.5 |
|  |  |  | - Presented in view 3 |
|  |  |  | - Used by "tourist gain"   Based on Based on Bossel’s Systems Zoo |
| tourist gain | auxiliary-Normal | dmnl/ month | *Gain of tourists per month.* |
|  |  |  | **Equation:**  DELAY1(attractiveness*TOURIST RATE*Tourist, 60) |
|  |  |  | - Presented in view 3 |
|  |  |  | - Used by "Tourists" |
| Tourist | Level-Normal | dmnl | *Number of tourists at time t* |
|  |  |  | **Equation:**  Tourist= INTEG ((tourist gain-loss), 1) |
|  |  |  | - Presented in view 3 |
|  |  |  | - Used by "Environmental stress" and "new investments" |
| Loss Rate | constant-Normal | dmnl | *Rate of tourist loss* |
|  |  |  | **Value**: 0.5 |
|  |  |  | - Presented in view 3 |
|  |  |  | - Used by "Loss"   Based on Based on Bossel’s Systems Zoo |
| Loss | constant-Normal | dmnl/  month | *Loss of tourists per month* |
|  |  |  | **Equation:**  Tourist*LOSS RATE |
|  |  |  | - Presented in view 3 |
|  |  |  | Used by "tourists" |
| Investment rate | constant-Normal | dmnl | *Rate at which investments are produced* |
|  |  |  | Value: 0.1 |
|  |  |  | - Presented in view 3 |
|  |  |  | - Used by "New investments" - Based on Based on Bossel’s Systems Zoo |
| New investments | auxiliary-Normal | dmnl/  month | *Investment to create new jobs and economic opportunities per month* |
|  |  |  | **Equation:**  new investments= DELAY FIXED (Tourist*INVESTMENT RATE, 36, 0) |
|  |  |  | - Presented in view 3 |
|  |  |  | - Used by "New jobs and economic opportunities" |
| New jobs and economic opportunities | level-Normal | dmnl | *New jobs and economic opportunities at time t* |
|  |  |  | **Equation:**  New jobs and economic opportunities= INTEG (new investments-loss of jobs, 1) |
|  |  |  | - Presented in view 3 |
|  |  |  | - Used by "profit" and "loss of jobs" |
| loss of jobs | auxiliary-Normal | dmnl/  month | Loss of jobs and economic opportunities per month |
|  |  |  | **Equation:**  Loss of jobs= New jobs and economic opportunities*loss of jobs rate |
|  |  |  | - Presented in view 3 |
|  |  |  | - Used by "New jobs and economic opportunities" |
| Loss of jobs rate | constant-Normal | dmnl | *Rate at which jobs and economic opportunities are lost* |
|  |  |  | **Value:** 0.1 |
|  |  |  | - Presented in view 3 |
|  |  |  | - Used by "loss of jobs"   Based on Based on Bossel’s Systems Zoo |
| Income | auxiliary-Normal | dmnl | *Economic benefit produced by new jobs and economic opportunities and agriculture.* |
|  |  |  | **Equation:**  Income= New jobs and economic opportunities+ (Mean productivity of extracted water*Units correction) |
|  |  |  | - Presented in view 3 |
|  |  |  | Used by "profit" |
| Mean productivity of extracted water | Auxiliary with Lookup | dmnl | *Productivity of extracted water (economic benefit produced by the exploitation of water resources).* |
|  |  |  | **Equation:**  Mean productivity of extracted water= WITH LOOKUP (GW extractions,([(0,0 (20,10)],(0,0),(1,0.2),(2,0.4),(3,0.6),(4,0.8),(5,1),(6,1.2),(7,1.4),(8,1.6),(9,1.8),(10,2),(11,2.2),(12,2.4),(13,2.6),(14,2.8),(15,3),(16,3.2 ),(17,3.4),(18,3.6),(19,3.8),(20,4) )) |
|  |  |  | - Presented in view 3 |
|  |  |  | - Used by "Income" |
| Profit | auxiliary-Normal | dmnl | *Economic profit produced by new jobs and economic opportunities and agriculture.* |
|  |  |  | **Equation:** Profit= income-(cost new infrastructure+(cost of extracting water*Units correction 2)) |
|  |  |  | - Presented in view 3 |
|  |  |  | Used by "pop increase" and "Environmental awareness" |
| Investment in infrastructure | auxiliary-Normal | dmnl/  month | *Investment in new infrastructures and services per month* |
|  |  |  | **Equation**  Investment in infrastructure= INVESTMENT RATE*profit |
|  |  |  | - Presented in view 3 |
|  |  |  | Used by "Infrastructure and services" |
| Infrastructure and services | level-Normal | dmnl | *Infrastructure and services at time t* |
|  |  |  | **Equation:**  infrastructure and services= INTEG (investment in infrastructure, 1) |
|  |  |  | - Presented in view 3 |
|  |  |  | Used by "cost of new infrastructure" and "pop increase" |
| cost of new infrastructure | auxiliary-Normal | dmnl | *Economic cost produced by the creation and maintenance of new infrastructures* |
|  |  |  | **Equation**  cost of new infrastructure=infrastructure and services*0.2 |
|  |  |  | - Presented in view 3 |
|  |  |  | Used by "Profit" |
| Cost of extracting water | Auxiliary with Lookup | dmnl | *Cost of extracting water from the aquifer. If the aquifer level is low, the cost of extracting water is higher.* |
|  |  |  | **Equation**  cost of extracting water= WITH LOOKUP (depth of wells,([(0,0)(39,10)],(0,0),(1,0.1),(2,0.2),(3,0.3),(4,0.4),(5,0.6),(6,0.7),(7,0.8),(8,0.9),(9,1),(10,1.1),(11,1.2),(12,1.3),(13,1.4),(14,1.5),(15,1.6),(16,1.7),(17,1.8),(18,1.9),(19,2),(20,2.1),(21,2.2),(22,2.3),(23,2.4),(24,2.5),(25,2.6),(26,2.7),(27,2.8),(28,2.9),(29,3),(30,3.1),(31,3.2),(32,3.3),(33,3.4),(34,3.5),(35,3.6),(36,3.7),(37,3.8),(38,3.9),(39,4) )) |
|  |  |  | - Presented in view 3 |
|  |  |  | - Used by "Profit" |
| pop increase | auxiliary-Normal | dmnl/ month | *Increase in rural population per month.* |
|  |  |  | **Equation**  Pop increase=infrastructure and services*profit*INMIGRATION RATE*rural population |
|  |  |  | - Presented in view 3 |
|  |  |  | Used by "rural population" |
| Rural population | level-Normal | dmnl | *Rural population at time t* |
|  |  |  | **Equation**  rural population= INTEG (pop increase-depopulation,1) |
|  |  |  | - Presented in view 3 |
|  |  |  | - Used by "pop increase", "depopulation" and "environmental degradation" |
| depopulation | auxiliary-Normal | dmnl/ month | *Loss of rural population per month* |
|  |  |  | **Equation**  depopulation=DEPOPULATION RATE*rural population |
|  |  |  | - Presented in view 3 |
|  |  |  | - Used by "Rural population" |
| depopulation rate | constant-Normal | dmnl | *Rate at which rural population decrease* |
|  |  |  | Value: 0.01 |
|  |  |  | - Presented in view 3 |
|  |  |  | - Used by "depopulation"   Based on Based on Bossel’s Systems Zoo |
| environmental stress | auxiliary-Normal | dmnl | *Environmental response to potential stressors such as noise, pollution and crowding that are exacerbated by the increase in tourist local population. The environmental stress could be reduced with high level of environmental awareness*. |
|  |  |  | **Equation**  Environmental stress= (rural population+4*Tourist)-Environmental awareness |
|  |  |  | - Presented in view 3 |
|  |  |  | - Used by "degradation" |
| Level of information/Citizens engagement | constant-Normal | dmnl | *Level of public information that is available for rural citizens and tourists. Civil engagement includes communities working together or individuals working alone in both political and non-political actions to protect nature.* |
|  |  |  | **Value:** 0.1 (low level of information/citizens engagement)-1(high level of information/citizens engagement) |
|  |  |  | - Presented in view 3 |
|  |  |  | - Used by "Environmental awareness"   Own elaboration |

***^1^** *Value Used in the model*

**References**

**[1]** Adekalu, K.O., Olorunfemi, I.A., Osunbitan, J.A., 2007. Grass mulching effect on infiltration, surface runoff and soil loss of three agricultural soils in Nigeria. Bioresour. Technol. 98, 912–917. https://doi.org/https://doi.org/10.1016/j.biortech.2006.02.044

**[2]** AEMET, 2020. Propiedades hidráulicas del suelo: capacidad de retención de agua. Madrid.

**[3]** Basche, A.D., DeLonge, M.S., 2019. Comparing infiltration rates in soils managed with conventional and alternative farming methods: A meta-analysis. PLoS One 14, e0215702–e0215702. https://doi.org/10.1371/journal.pone.0215702

**[4]** Calatrava, J., Mayor, B., Martínez-Granados, D., 2019: “DELIVERABLE 6.3 DEMO Insurance value assessment report- Part 1: SPAIN – Medina del Campo”. EU Horizon 2020 NAIAD Project, Grant Agreement N°730497

**[5]** CHD, 2013. Informe sobre zonificacion de las masas de agua subterranea Tordesillas, Los Arenales, Medina del Campo y Tierra del Vino y propuesta de normas de otorgamiento de concesiones y autorizaciones en dichas masas.

**[6]** IGME, 2015. Actividad 2:Apoyo a la caracterización adicional de las masas de agua subterránea en riesgo de no cumplir los objetivos medioambientales en 2015- Demarcación Hidrográfica del Duero MASA DE AGUA SUBTERRÁNEA 47 Medina del Campo.

**[7]** IGME, 2012. Las AGUAS subterráneas en la planificación hidrogeológica. Madrid.

**[8]** IGME, 2010. Identificación y caracterización de la interrelación que se presenta entre aguas subterráneas, cursos fluviales, descarga por manantiales, zonas húmedas y otros ecosistemas naturales de especial interés hídrico.

**[9]** IGME, 2008. Integración de las masas de aguas subterráneas en el modelo de gestión de la cuenca hidrográfica del duero. determinación de los parámetros de simulación (coeficientes de agotamiento).

**[10]** Ministerio de Agricultura, P. y A., 2019. Encuesta sobre Superficies y Rendimientos Cultivos (ESYRCE). Encuesta de Marco de Áreas de España. [WWW Document]. URL https://www.mapa.gob.es/es/estadistica/temas/estadisticas-agrarias/agricultura/esyrce/ (accessed 6.1.20).

**[11]** Plan de monitorización de los cultivos de regadío en Castilla y León resultados de la encuesta de cultivos con datos acumulados de las campañas agrícolas 2011-2018, 2020 . Valladolid.

**[12]** Porée, Lucille. Evolución de las zonas riparias de los ríos Trabancos, Zapardiel y Adaja (cuenca del Duero) entre 1956 y la actualidad. Universidad Politécnica de Cartagena, 2019.

**[13]** Schosinsky, G., 2006. Cálculo de la recarga potencial de acuíferos mediante un balance hídrico de suelos. Rev. Geológica América Cent. https://doi.org/10.15517/rgac.v0i34-35.4223

**[14]** Sithole, N.J., Magwaza, L.S., Thibaud, G.R., 2019. Long-term impact of no-till conservation agriculture and N-fertilizer on soil aggregate stability, infiltration and distribution of C in different size fractions. Soil Tillage Res. 190, 147–156. https://doi.org/https://doi.org/10.1016/j.still.2019.03.004

**[15]** Thematic Brief 5-Water Use Efficiency: Brief prepared for the Entry phase of the project: Strengthening Agricultural Water Efficiency and Productivity on the African and Global Level, 2016.

**[16]** Thierfelder, C., Wall, P.C., 2010. Investigating Conservation Agriculture (CA) Systems in Zambia and Zimbabwe to Mitigate Future Effects of Climate Change. J. Crop Improv. 24, 113–121. https://doi.org/10.1080/15427520903558484

**[17]** Thierfelder, C., Wall, P.C., 2009. Effects of conservation agriculture techniques on infiltration and soil water content in Zambia and Zimbabwe. Soil Tillage Res. 105, 217–227. https://doi.org/https://doi.org/10.1016/j.still.2009.07.007

**[18]** Van der Keur, P. et al., 2018: “DELIVERABLE 6.2 From hazards to risk: models for the DEMOs”. EU Horizon 2020 NAIAD Project, Grant Agreement N°730497.Should you wish to use or refer to individual part/-s of the report, please cite according to the suggestion within the individual report

**[19]** Water conservation factsheet-soil water storage capacity and available soil moisture, 2015. Abbotsford, B.C.
