# Supplementary material for: Assessing the long-term effectiveness of Nature-Based Solutions under different climate change scenarios
Source: Sci Total Environ. 2021 Nov 10;794:148515. doi: 10.1016/j.scitotenv.2021.148515 (PMC8434435; doi:10.1016/j.scitotenv.2021.148515)
Supplement: Appendix C — Table 1C. List of climate model used in the evaluation and multi-model simulations (Jacob et al., 2020). [file mmc3.docx]

**Appendix C**

*Table 1C. List of climate model used in the evaluation and multi-model simulations (Jacobs 2020)*

| Driving Model | Run type | Regional climate model (RCM) |
| --- | --- | --- |
| ICHEC-EC-EARTH | simulation rcp26 | SMHI-RCA4 |
| MOHC-HadGEM2-ES | simulation rcp26 | KNMI-RACMO22E |
| MPI-M-MPI-ESM-L | simulation rcp26 | MPI-CSC-REMO2009 |
| MPI-M-MPI-ESM-LR | simulation rcp26 | MPI-CSC-REMO2009 |
| ICHEC-EC-EARTH | simulation rcp45 | DMI-HIRHAM5 |
| ICHEC-EC-EARTH | simulation rcp45 | CLMcom-CCLM4-8-17 |
| ICHEC-EC-EARTH | simulation rcp45 | SMHI-RCA4 |
| IPSL-IPSL-CM5A-MR | simulation rcp45 | SMHI-RCA4_v1 |
| MOHC-HadGEM2-ES | simulation rcp45 | CLMcom-CCLM4-8-17 |
| MOHC-HadGEM2-ES | simulation rcp45 | KNMI-RACMO22E |
| MOHC-HadGEM2-ES | simulation rcp45 | SMHI-RCA4 |
| MPI-M-MPI-ESM-LR | simulation rcp45 | CLMcom-CCLM4-8-17 |
| MPI-M-MPI-ESM-LR | simulation rcp45 | MPI-CSC-REMO2009 |
| MPI-M-MPI-ESM-LR | simulation rcp45 | SMHI-RCA4 |
| MPI-M-MPI-ESM-LR | simulation rcp45 | MPI-CSC-REMO2009 |
| ICHEC-EC-EARTH | simulation rcp85 | KNMI-RACMO22E |
| ICHEC-EC-EARTH | simulation rcp85 | DMI-HIRHAM5 |
| ICHEC-EC-EARTH | simulation rcp85 | CLMcom-CCLM4 |
| ICHEC-EC-EARTH | simulation rcp85 | SMHI-RCA4 |
| IPSL-IPSL-CM5A-MR | simulation rcp85 | SMHI-RCA4 |
| MOHC-HadGEM2-ES | simulation rcp85 | CLMcom-CCLM4-8-17 |
| MOHC-HadGEM2-ES | simulation rcp85 | KNMI-RACMO22E |
| MOHC-HadGEM2-ES | simulation rcp85 | SMHI-RCA4 |
| MPI-M-MPI-ESM-LR | simulation rcp85 | CLMcom-CCLM4-8-17 |
| MPI-M-MPI-ESM-LR | simulation rcp85 | MPI-CSC-REMO2009 |
| MPI-M-MPI-ESM-LR | simulation rcp85 | SMHI-RCA4 |
| MPI-M-MPI-ESM-LR | simulation rcp85 | MPI-CSC-REMO2009 |

**Bibliography**

Jacob, D.; Teichmann, C.; Sobolowski, S.; Katragkou, E.; Anders, I.; Belda, M.; Benestad, R.; Boberg, F.; Buonomo, E.; Cardoso, R. M.; Casanueva, A.; Christensen, O. B.; Christensen, J. H.; Coppola, E.; Cruz, L. D.; Davin, E. L.; Dobler, A.; Domínguez, M.; Fealy, R.; Fernandez, J.; Gaertner, M. A.; García-Díez, M.; Giorgi, F.; Gobiet, A.; Goergen, K.; Gómez-Navarro, J. J.; Alemán, J. J. G.; Gutiérrez, C.; Gutiérrez, J. M.; Güttler, I.; Haensler, A.; Halenka, T.; Jerez, S.; Jiménez-Guerrero, P.; Jones, R. G.; Keuler, K.; Kjellström, E.; Knist, S.; Kotlarski, S.; Maraun, D.; van Meijgaard, E.; Mercogliano, P.; Montávez, J. P.; Navarra, A.; Nikulin, G.; de Noblet-Ducoudré, N.; Panitz, H.-J.; Pfeifer, S.; Piazza, M.; Pichelli, E.; Pietikäinen, J.-P.; Prein, A. F.; Preuschmann, S.; Rechid, D.; Rockel, B.; Romera, R.; Sánchez, E.; Sieck, K.; Soares, P. M. M.; Somot, S.; Srnec, L.; Sørland, S. L.; Termonia, P.; Truhetz, H.; Vautard, R.; Warrach-Sagi, K. & Wulfmeyer, V. Regional climate downscaling over Europe: perspectives from the EURO-CORDEX community Regional Environmental Change, Springer Science and Business Media LLC, 2020, 20
